# Lysosomal Cathepsin S Escape Facilitates Near Infrared Light‐Triggered Pyroptosis Via an Antibody‐Indocyanine Green Conjugate

**DOI:** 10.1002/advs.202504851

**Published:** 2025-06-20

**Authors:** Fan Chen, Xue‐Fei Tian, Teng Yang, Yu‐Jie Dai, Da‐Yuan Chen, Hong‐Bo Chen, Takaya Shimura, Xin‐Fang Li, Chu‐Lin Sha, Qing Ji, Jun Cao, Mei‐Yu Fang, Jin‐Biao Shang, Jian‐Min Fang, Ye Lu, Wei‐Hui Zheng, Peng Guo, Wei‐Hong Tan

**Affiliations:** ^1^ Zhejiang Cancer Hospital Hangzhou Institute of Medicine (HIM) Chinese Academy of Sciences Hangzhou Zhejiang 310018 China; ^2^ Molecular Science and Biomedicine Laboratory (MBL) State Key Laboratory of Chemo/Biosensing and Chemometrics College of Chemistry and Chemical Engineering College of Biology Aptamer Engineering Center of Hunan Province Hunan University Changsha Hunan 410082 China; ^3^ School of Molecular Medicine Hangzhou Institute for Advanced Study UCAS Hangzhou 310024 China; ^4^ College of Pharmaceutical Science Zhejiang University of Technology Hangzhou Zhejiang 310014 China; ^5^ MabPlex International Yantai Shandong 264006 China; ^6^ Guilin University of Electronic Technology Guilin Guangxi 541004 China; ^7^ Department of Gastroenterology and Metabolism Nagoya City University Graduate School of Medical Sciences Nagoya 467‐8601 Japan; ^8^ Department of Head and Neck and Rare Oncology Zhejiang Cancer Hospital Hangzhou Zhejiang 310022 China; ^9^ School of Life Science and Technology Tongji University Shanghai 200092 China

**Keywords:** anti‐tumor immunity, antibody‐drug conjugate, pyroptosis

## Abstract

Pyroptosis is a proinflammatory programmed cell death (PCD) that is causally linked to antitumor immune responses, but the therapeutic potential of pyroptosis has been limited by the lack of tumor‐specific and controllable inducers. Here, it is reported that tumor‐specific pyroptosis can be spatiotemporally triggered via near‐infrared light (NIR‐pyroptosis) by using an antibody‐bound indocyanine green (ICG), a clinically approved and nontoxic fluorescent dye. Mechanistically, the key molecular steps are identified by which antibody‐bound ICG generates excessive reactive oxygen species (ROS) within lysosomes after internalization, leading to lysosomal membrane damage and the cytosolic release of cathepsin S (CTSS), which cleaves gasdermin D (GSDMD), IL‐18, and IL‐1β independently of caspase‐1, and thereby induces pyroptosis, while other cathepsin family members fail to cleave GSDMD. Functionally, in both ICAM1+ and HER2+ solid tumors, antibody‐bound ICG‐mediated NIR‐pyroptosis triggers potent and durable antitumor immune responses through the release of proinflammatory cytokines. Furthermore, NIR‐pyroptosis synergize with anti‐PD‐1 therapy by activating adaptive immune cells via upregulated IFN‐γ secretion. The findings identify CTSS as a novel enzyme for GSDMD cleavage and establish NIR‐pyroptosis as a non‐apoptotic anticancer modality, providing a promising opportunity to overcome apoptosis resistance in current cancer therapies.

## Graphical abstract

ICAM1 antibody‐bound ICG triggers tumor‐specific, caspase‐1‐independent pyroptosis via CTSS‐mediated GSDMD cleavage, activates antitumor immunity, and synergizes with anti–PD‐1 therapy, offering a non‐apoptotic strategy to overcome resistance in cancer treatment.

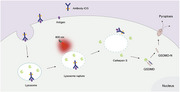

## 1 Introduction

To date, most anticancer therapies (e.g., chemotherapy, radiotherapy, and immunotherapy) induce apoptosis, a non‐inflammatory programmed cell death (PCD), in cancer cells, but resistance to such treatments is common owing to the evasion of apoptosis machinery in response to cellular stress.^[^ ^1^, ^2^ ^]^ To overcome apoptotic resistance, non‐apoptotic models of PCDs (e.g., pyroptosis, necrosis, ferroptosis, and autophagy) have recently gained increasing attention, since they hold the promise of bypassing the apoptosis pathway to induce cancer cell death.^[^ ^3^, ^4^, ^5^, ^6^ ^]^ Among these non‐apoptotic PCDs, pyroptosis holds tremendous potential in enhancing antitumor immunity by its ability to prime antitumor immune responses by releasing cellular content and inflammatory factors (IL‐1β and IL‐18), thereby transforming cold solid tumors into hot tumors.^[^ ^7^ ^]^ Recent studies have demonstrated that the robust antitumor immune response triggered by pyroptosis may amplify the response rate of PD‐1 blockade therapy.^[^ ^8^ ^]^Consequently, the exploitation and development of an efficient combination strategy to induce tumor‐specific pyroptosis, while maximizing the response rates to immune checkpoint inhibitors (ICIs), could present new opportunities in cancer treatment.

The current development of pyroptosis‐based anticancer therapy has been limited by the severe lack of inducers to trigger pyroptotic cell death in a tumor‐specific and controllable manner. Several recent studies have indicated that small molecules, iron, modified viruses as viral vectors, chemotherapeutic agents, and nanomedicines^[^ ^9^ ^]^ can induce GSDMD/GSDME‐mediated pyroptosis in various malignant cancers.^[^ ^10^ ^]^ Nevertheless, most existing pyroptotic inducers lack specificity and potency for particular cell types, causing undesirable adverse effects.^[^ ^11^ ^]^ Therefore, an urgent and unmet need remains for developing tumor‐selective pyroptosis inducers.

Antibody‐drug conjugates (ADCs) are considered as a new class of targeted therapeutics for many hematological malignancies and solid tumors.^[^ ^12^ ^]^ Their potent clinical efficacies have led to 15 ADCs approved within the past two decades, with over 200 additional ADCs entering clinical trials.^[^ ^13^, ^14^ ^]^ To date, 14 out of 15 clinically‐approved ADCs primarily act on inducing apoptotic cancer cell death through cytotoxic warheads (e.g., microtubule or topoisomerase inhibitors) conjugated to a tumor‐homing monoclonal antibody.^[^ ^15^ ^]^ Notably, cetuximab sarotalocan, an EGFR antibody‐conjugated IR700, is the only clinically‐approved ADC employing non‐apoptotic PCD for head and neck cancer.^[^ ^16^ ^]^ By harnessing its photo‐switchable warhead, IR700, a silica‐phthalocyanine dye, cetuximab sarotalocan changes molecular hydrophobicity on cancer cell membranes upon absorbing NIR photons, leading to cell membrane rupture and subsequent immunogenic cell death, which is also known as near‐infrared photoimmunotherapy (NIR‐PIT).^[^ ^17^, ^18^, ^19^ ^]^ However, its underlying molecular mechanism(s) remain(s) largely unknown.

Inspired by ADCs, we herein explored ICG, a non‐toxic, liver‐metabolized small‐molecular fluorescent dye (molecular weight, MW, 775D), as a novel NIR‐pyroptosis warhead to develop ADCs for anaplastic thyroid cancer (ATC), one of the most aggressive solid tumors with a 1‐y survival rate as low as 20%, and patients with ATC rarely survive more than 2 years after diagnosis.^[^ ^20^ ^]^ ICG has been widely used for imaging‐guided surgery,^[^ ^21^, ^22^ ^]^ ophthalmic angiography,^[^ ^23^ ^]^ lymph node mapping,^[^ ^24^, ^25^ ^]^ blood flow evaluation,^[^ ^26^, ^27^ ^]^ and liver function assessment,^[^ ^28^, ^29^ ^]^ owing to its outstanding safety profiles and NIR fluorescence properties. Moreover, ICG has been utilized as a photosensitizer in photodynamic therapy (PDT), as well as a photothermal agent in photothermal therapy (PTT), noting that the mechanisms of action (MoAs) of both PDT and PTT rely on DNA damage‐induced cancer cell apoptosis.^[^ ^30^, ^31^ ^]^ To date, the function of ICG as an NIR‐triggered pyroptotic inducer has not been explored.

Interestingly, tumor cells respond differently to the same drugs with different molecular weights. An outstanding case is an albumin‐bound paclitaxel, the pharmacological behavior of which is distinctly different from that of paclitaxel in free form.^[^ ^32^ ^]^ Inspired by this phenomenon, we asked if antibody‐bound ICG (MW, 150kD), in comparison to small molecular ICG in free form (MW, 775D), could potently induce a pyroptosis‐like phenotype in cancer cells following NIR light irradiation. To elucidate its underlying biological mechanism, we found that antibody‐bound ICG enters cancer cells through antigen‐mediated endocytosis and produces massive ROS within intracellular lysosomes under NIR light irradiation. High concentrations of localized ROS in lysosomes efficiently damage organelle membranes, leading to the release of lysosomal proteases into the cytosol. Among six cathepsin family members, cathepsin S (CTSS) was uniquely identified as the lysosomal protease capable of effectively cleaving GSDMD, IL‐1β, and IL‐18 independently of caspase‐1. This cleavage triggers GSDMD‐N‐mediated membrane pore formation and the release of pro‐inflammatory cytokines, ultimately inducing tumor cell pyroptosis. CTSS is a unique cysteine protease distinguished by its ability to remain active at neutral pH, unlike most family members.^[^ ^33^ ^]^ This biochemical trait enhances its potential involvement in extra‐lysosomal proteolytic activities such as MHC Class II antigen presentation, by cleaving the invariant chain.^[^ ^34^ ^]^ Notably, our findings represent the first report implicating the role of CTSS in cleaving key proteins involved in the pyroptosis process independently of caspase‐1. This NIR‐pyroptosis ADC, featuring a novel MoA, is distinctly different from conventional chemotherapy and radiotherapy that target DNA‐damage response (DDR). Instead, NIR‐pyroptosis uses NIR light to spatiotemporally induce proinflammatory pyroptotic cell death and, as a result, boost antitumor immunity with long‐lasting immune memory, highlighting a new opportunity to overcome apoptosis resistance and sensitize checkpoint immunotherapy in solid tumor treatments.

## 2 Results

### 2.1 Identification of Optimal ATC Target for NIR‐Pyroptosis

**Figure 1:**
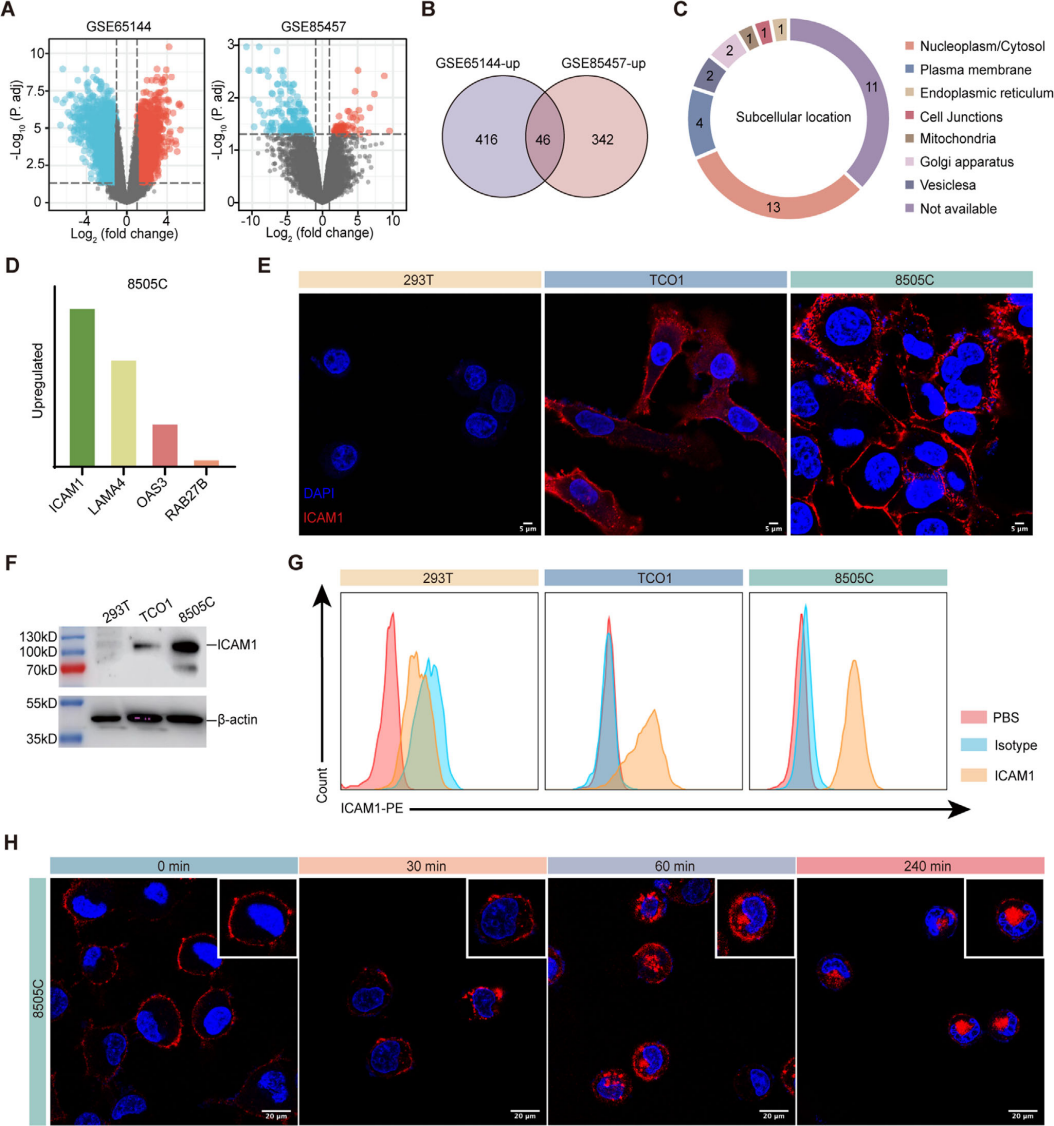
ICAM1 was identified as a suitable target for ATC. A) Volcano plot of DEGs from two ATC‐related datasets. B) Venn diagram showing the overlaps of upregulated genes between the selected datasets for ATC. C) Subcellular localization of 46 candidate genes encoding proteins. D) The expression of 4 upregulated surface proteins in human ATC cells (8505C). E) Immunofluorescence staining of ICAM1 on human ATC cells (TCO1 and 8505C) and normal 293T cells (Scale bars, 5 µm). F) Western blot analysis of ICAM1 on human ATC cells (TCO1 and 8505C) and normal 293T cells. G) Surface expression of ICAM1 on human ATC cells (TCO1 and 8505C) was compared against that of normal 293T cells by flow cytometry using PE‐labeled antibody. Nontargeting IgG was used as a control. H) Representative images showing cellular internalization of ICAM1 antibodies in human ATC cells (8505C) (Scale bars, 20 µm).

In the absence of a clinically effective ATC target for ADC, we first performed an unbiased and quantitative screening to identify an optimal molecular target that distinguishes ATC tumors from normal tissues. We employed publicly available databases to screen potential cell membrane protein targets. Two datasets relevant to ATC (GSE65144 and GSE85457) were retrieved from the Gene Expression Omnibus (GEO) database (Table S1, Supporting Information). Applying |Log_2_(FC)| > 2 and P < 0.05 as threshold criteria, we identified 462 upregulated genes in GSE65144 and 388 upregulated genes in GSE85457 (**Figure** **1**A). Venn diagram analysis further revealed 46 commonly upregulated genes in both datasets, among which 11 were identified as long non‐coding RNAs (lncRNAs) and excluded from further analysis (Figure 1B). The remaining 35 genes were selected as target candidates for subsequent investigation. In Figure 1C, we queried the Human Protein Atlas database (https://www.proteinatlas.org) to analyze the subcellular localization of these target gene‐encoded proteins. We found 13 targets to be localized to the cytoplasm or nucleus, while seven were found in organelles, such as mitochondria and Golgi apparatus. Remarkably, only four targets were located on the plasma membrane, including ICAM1, Laminin Subunit Alpha 4 (LAMA4), 2',5'‐oligoadenylate synthase 3 (OAS3), and Rab7b, respectively. We compared the mRNA expression of these four cell membrane antigens in an established human ATC cell line (8505C) and found that ICAM1 exhibited the highest expression (Figure 1D).

We further validated ICAM1 overexpression at protein levels in two human ATC cell lines (TCO1 and 8505C), along with normal 293T cells, using Western blotting (WB), immunofluorescence (IF) staining, and flow cytometry. Results consistently confirmed the overexpression of ICAM1 in ATC cell lines, while it was absent in normal 293T cells, with ICAM1 predominantly located on the plasma membranes (Figure 1E–G). Considering that cellular internalization is another key factor for ADC efficacy, we next used IF‐staining to assess whether ICAM1 ADCs could efficiently penetrate ATC. Confocal fluorescence images revealed that the fluorescence of PE‐conjugated ICAM1 antibodies was predominantly localized to the cytoplasmic membrane of 8505C cells during the initial 30 min of incubation. They were gradually trafficked into the endosomes/lysosomes of 8505C cells through receptor‐mediated endocytosis after 1‐h incubation (Figure 1H). These results strongly support that ICAM1 is a promising ATC target for NIR‐pyroptosis treatment.

### 2.2 Design, Synthesis, and Characterization of NIR‐Pyroptosis ADCs

**Figure 2:**
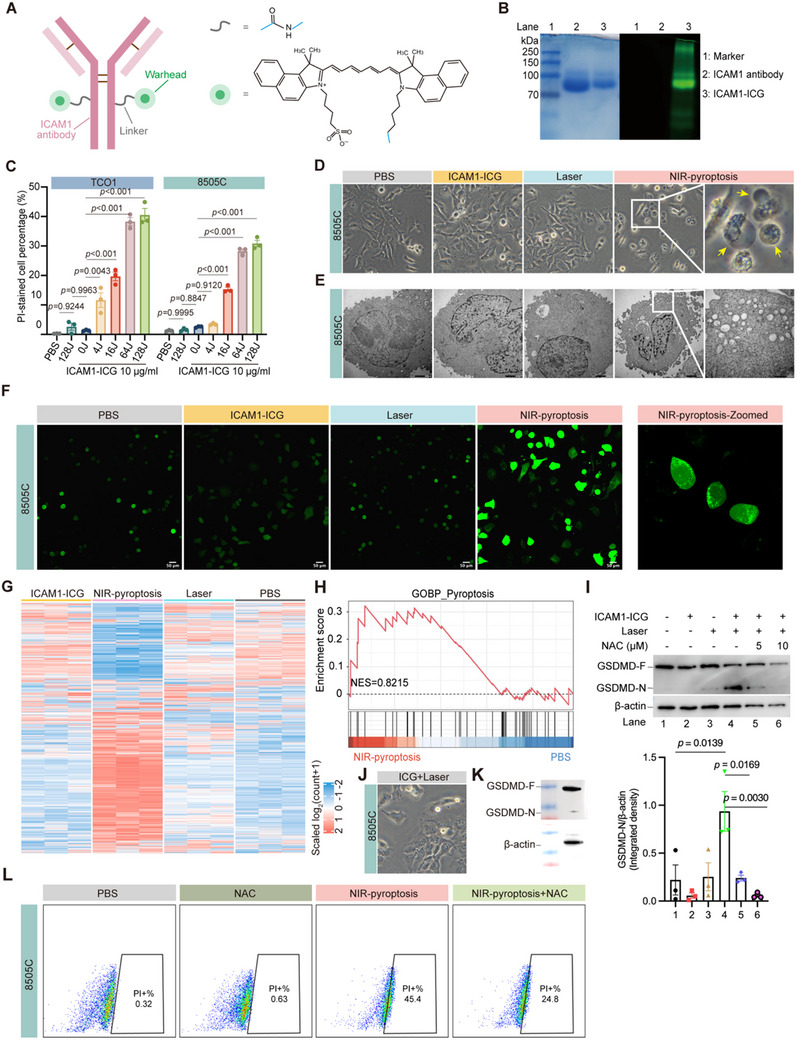
Pyroptotic effects of ICAM1‐ICG under NIR light irradiation on ATC cells. A) Schematic illustration of an ICAM1‐ICG (Left). Chemical structures of ADC linkers and warheads used in ICAM1‐ICG (Right). B) Validation of ICAM1‐ICG by SDS‐PAGE (left: Colloidal Blue staining, right: fluorescence). Diluted ICAM1 mAb was used as a control. C)The in vitro cytotoxicity of ICAM1‐ICG on ATC cells with a serial dose of NIR light irradiation, using propidium iodide (PI) staining. D) Representative phase‐contrast cell images of ATC cells treated with NIR‐pyroptosis. Arrows mark cells that show pyroptotic morphology. E) Representative TEM images of 8505C cells treated with NIR‐pyroptosis (Scale bars, 2 µm). F) Representative fluorescence images of intracellular ROS detection using DCFH‐DA staining (Scale bars, 50 µm). G) Heatmap of top 5000 genes with significant expression in 8505C cells treated with ICAM1‐ICG, NIR‐pyroptosis, Laser, or PBS, respectively, followed by RNA‐seq analysis (n = 3). H) GSEA of the pyroptosis signaling pathway, comparing the NIR‐pyroptosis and PBS groups. I) Western blot analysis of GSDMD cleavage in 8505C cells with different treatments (Up). NAC was used to eliminate intracellular ROS. Quantitative analysis of GSDMD‐N relative expression (Down) (n = 3, mean ± SEM). J) Representative phase‐contrast cell images of 8505C cells treated with solvent‐based ICG under NIR light. K) Western blot analysis of GSDMD cleavage in 8505C cells treated with solvent‐based ICG under NIR light. L) Flow cytometry analysis of NIR‐pyroptosis cytotoxicity after adding NAC. In C) and I), data are presented as mean ± SEM, and differences between each group are determined by One‐way ANOVA.

To design an optimal ATC‐targeted NIR‐pyroptosis ADC, we covalently conjugated ICAM1 antibody with ICG, a clinically‐used NIR fluorescent dye, as a warhead. Its excitation wavelength is ≈600–950 nm, allowing for penetration into relatively deeper tissues. Small molecular ICG in free form has a short circulation half‐life (3‐4 min) and lacks tumor selectivity.^[^ ^35^, ^36^ ^]^ However, taking advantage of the tumor‐targeting capability of ICAM1 monoclonal antibody, we designed and synthesized an ICAM1 antibody‐bound ICG (ICAM1‐ICG, **Figure** **2**A) by covalently conjugating ICG‐NHS ester with the amine groups of lysine in the ICAM1 antibody. The as‐synthesized ICAM1‐ICG was characterized by SDS‐PAGE. Both ICAM1‐ICG and the unconjugated ICAM1 monoclonal antibody showed similar molecular weights, but ICAM1‐ICG exhibited strong NIR fluorescence from the antibody‐bound ICGs in comparison with the unconjugated ICAM1 antibody (Figure 2B). The drug‐to‐antibody ratio (DAR) value was determined by MALDI‐TOF MS as 2.03 (Figure S1A, Supporting Information). Furthermore, we quantitatively assessed the in vitro cytotoxicity of ICAM1‐ICG by NIR irradiation against two human ATC cell lines. Under a serial dose of NIR light irradiation, ICAM1‐ICG exhibited a dose‐dependent cytotoxicity against ATC cells. In contrast, no cytotoxicity was observed for groups with either NIR irradiation or ICAM1‐ICG alone (Figure 2C). Collectively, these in vitro efficacy data support further investigation of ICAM1‐ICG under NIR light in ATC in vivo models.

### 2.3 NIR Light Triggers Tumor‐Specific Pyroptosis via ICAM1‐ICG

Strikingly, distinct morphological changes were observed in ICAM1‐ICG‐treated ATC cells under NIR irradiation. Such changes were characterized by cell body swelling into balloon‐like vesicles, followed by cell membrane ruptures, resembling the typical features of cellular pyroptosis reported in the literature (Figure 2D and Figure S2A, Supporting Information).^[^ ^37^, ^38^, ^39^ ^]^ These pyroptosis‐like morphological changes were further confirmed under transmission electron microscopy (TEM), including nuclear condensation and the appearance of multiple vacuoles near the cell membrane under ICAM1‐ICG+NIR treatment (Figure 2E). Since the NIR radiation product of ICG is mainly ROS,^[^ ^40^, ^41^ ^]^ we next investigated the level of ROS in ICAM1‐ICG‐mediated pyroptosis of ATC cells. As shown in Figure 2F and Figure S2D (Supporting Information), we found that NIR light significantly upregulates ROS levels in the endosomes/lysosomes of ICAM1‐ICG‐treated ATC cells.

To decipher the MoA of ICAM1‐ICG, we performed RNA‐seq transcriptomic analysis of ATC cells under different treatments. By screening the top 5000 differentially expressed genes (DEGs), we found specific expression characteristics in the ICAM1‐ICG+NIR group (NIR‐pyroptosis) compared to the other groups (Figure 2G). Gene set enrichment analysis (GSEA) showed that the cellular pyroptosis signaling pathway was enriched in the NIR‐pyroptosis group in comparison with PBS (Figure 2H). To validate these transcriptomic findings at protein levels, Western blot analysis showed that a dosage of up to 5 µg mL^−1^ ICAM1‐ICG was sufficient to trigger cleavage of the crucial pore‐forming protein GSDMD, leading to effective cell pyroptosis (Figure S2B, Supporting Information). Meanwhile, a significant increase in the level of cleaved GSDMD‐N expression in ATC cells after ICAM1‐ICG‐mediated NIR‐pyroptosis (Figure 2I and Figure S2C, Supporting Information). Interestingly, we attempted to characterize the protein expression of caspase‐1, a classical GSDMD‐cleaving enzyme. However, the active form of caspase‐1 was undetectable, suggesting that an alternative enzyme may be responsible for mediating NIR‐pyroptosis independently of caspase‐1 (Figure S2E, Supporting Information). Moreover, small molecular ICG in free form under NIR light did not induce any pyroptotic cell death in ATC cells (Figure 2J,K).

The association between ROS and pyroptosis at the molecular level has been previously reported.^[^ ^42^ ^]^ Specifically, it was found that intracellular ROS effectively activates recognition receptors (NLRP1/3) to form inflammasome complexes and subsequently triggers GSDMD‐mediated pyroptosis.^[^ ^42^ ^]^ Therefore, we asked if ICAM1‐ICG‐generated ROS might be the predominant contributor of NIR light‐induced pyroptosis in our study. To address this question, we used N‐acetyl‐L‐cysteine (NAC), an established ROS scavenger, to block ROS production. We found that NAC treatment potently blocked GSDMD cleavage by 93.62% (Figure 2I). Furthermore, FACS analysis showed that NAC treatment rescued NIR‐pyroptosis‐mediated ATC cell death by 45.48% (Figure 2L). Collectively, the above biomechanistic results reveal that ICAM1‐ICG, rather than free ICG, under NIR light can, indeed, induce tumor‐specific pyroptosis.

### 2.4 Lysosomal CTSS Escape Facilitates GSDMD Cleavage in NIR‐Pyroptosis

**Figure 3:**
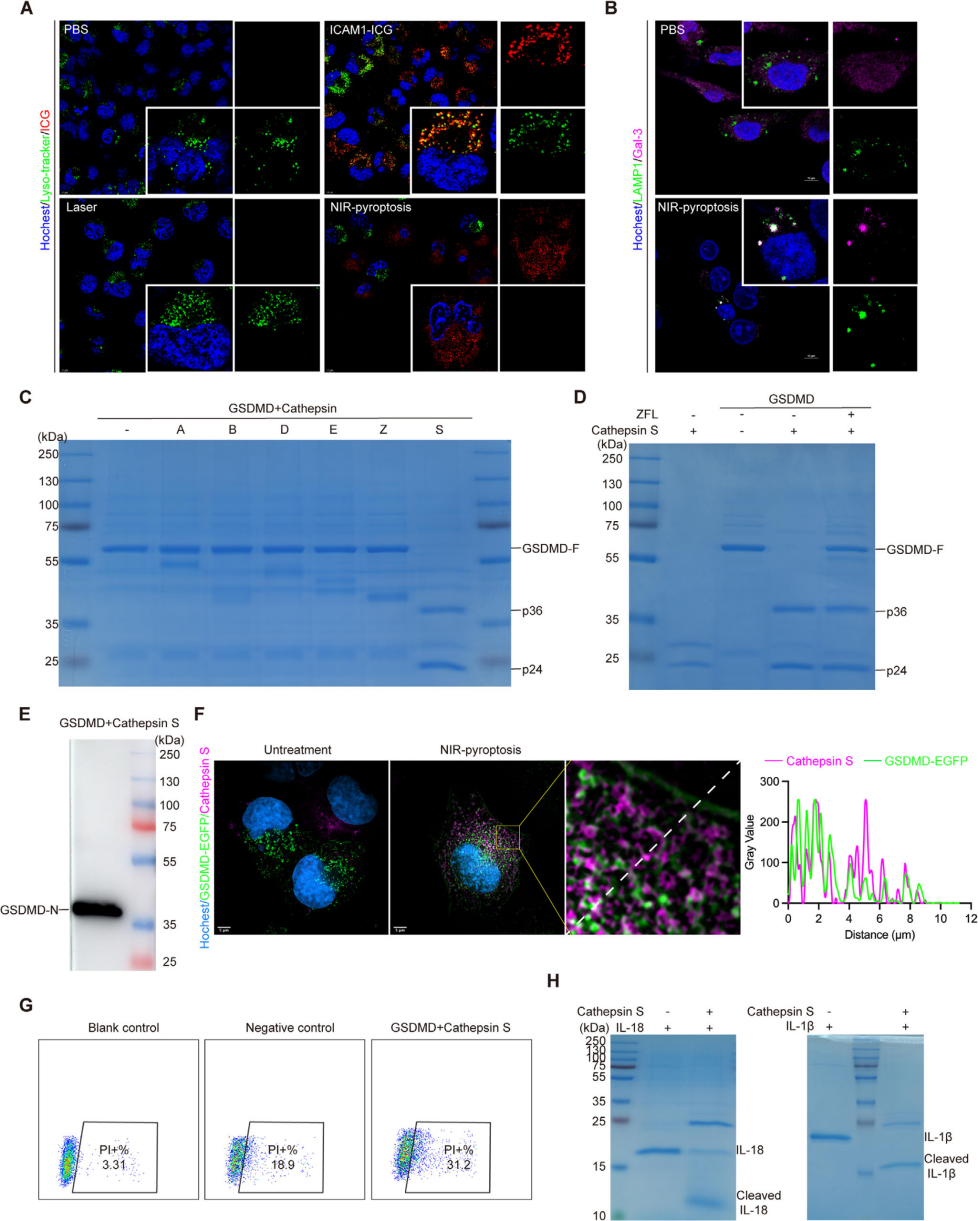
Recombinant GSDMD cleavage by cathepsin S. A) Confocal immunofluorescence images of lysosomes and ICAM1‐ICG after NIR‐pyroptosis (Scale bars, 10 µm). B) Confocal immunofluorescence images of LAMP1 and Gal‐3 after NIR‐pyroptosis (Scale bars, 10 µm). C) In vitro cleavage of GSDMD by recombinant cathepsin. D) Inhibitory effect of cathepsin S‐specific inhibitor on cathepsin S mediated cleavage of GSDMD. E) Western blot analysis of GSDMD cleavage mediated by cathepsin S. F) HiS‐SIM immunofluorescence images of GSDMD and cathepsin S in 8505C‐GSDMD‐EGFP cells after NIR‐pyroptosis (Scale bars, 5 µm). G) PI staining analysis after co‐expression of cathepsin S and GSDMD in 293T cells. H) In vitro cleavage of IL‐18 and IL‐1β by recombinant cathepsin S.

In our study, the internalized drug ICAM1‐ICG was observed to co‐localize with lysosomes (**Figure** **3**A). Following NIR irradiation, the green lysosomal probe signal disappeared, while the red fluorescence of ICAM1‐ICG transitioned from a punctate pattern to a diffuse distribution throughout the cytoplasm (Figure 3A), indicating that NIR‐pyroptosis likely causes lysosomal damage. Galectin‐3 (Gal‐3), recently identified as a marker of membrane damage,^[^ ^43^ ^]^ was used to further investigate this phenomenon. Under normal conditions, Gal‐3 is distributed across the cytoplasm and nucleus, with an affinity for β‐galactosides. Glycosylated compounds containing β‐galactosides are typically localized to the cell surface, endosomes, Golgi apparatus, and post‐Golgi secretory compartments. Gal‐3 can only bind to glycoproteins within these compartments when the lysosome membrane is disrupted.^[^ ^44^ ^]^ Further co‐localization analysis of LAMP‐1 and Gal‐3 provided additional evidence of lysosomal damage (Figure 3B), supporting the hypothesis that lysosomal disruption facilitates the release of lysosomal proteases (e.g., CTSS) into the cytoplasm, enabling subsequent GSDMD cleavage.

Considering that lysosomal damage results in the cytosolic release of various hydrolytic enzymes, we hypothesized that certain lysosomal hydrolases (e.g., Cathepsins) released into the cytoplasm may contribute to the cleavage of GSDMD. However, to date, none of the Cathepsin family members have been reported to directly cleave GSDMD and induce pyroptosis. To test this hypothesis, we evaluated the cleavage activity of six recombinant cathepsins on recombinant GSDMD protein (Figure 3C). The results showed that only CTSS could cleave GSDMD, leading to the disappearance of its full‐length form and the generation of ≈36 kD and ≈24 kD fragments, likely specific to CTSS activity. This exclusive GSDMD cleavage activity of CTSS is attributed to its unique ability to retain enzymatic activity across a broad pH range, from the acidic environment of lysosomes to the neutral pH of the cytoplasm.^[^ ^33^ ^]^ Addition of the cathepsin S‐specific inhibitor ZFL partially restored the full‐length GSDMD, underscoring the critical role of cathepsin S in this process. Immunoblotting analysis further confirmed the presence of the cleaved GSDMD‐N domain (Figure 3E), providing additional evidence of CTSS‐mediated GSDMD cleavage following lysosomal damage.

To further explore the relationship between GSDMD and CTSS, we established stable GSDMD‐EGFP‐expressing 8505C cell lines. Following NIR‐pyroptosis, significant co‐localization of GSDMD and CTSS was observed within the cells (Figure 3F). In GSDMD‐negative 293T cells co‐expressing CTSS and GSDMD, the cell death rate increased by 65% (from 18.9% to 31.2%) compared to the control group (Figure 3G). Furthermore, we found that CTSS cleaved both IL‐18 and IL‐1β, resulting in the near‐complete disappearance of their full‐length forms and the generation of smaller cleavage fragments, resembling a highly similar pattern from the classical caspase‐1‐mediated cleavage of these cytokines (Figure 3H). In summary, our findings reveal a novel mechanism in which CTSS, released from lysosomes during NIR‐pyroptosis, cleaves GSDMD in a similar but independent way of caspase‐1, and plays a critical role in the induction of NIR‐pyroptosis.

### 2.5 Antitumor Efficacy of ICAM1‐ICG‐Mediated NIR‐Pyroptosis In Vivo

**Figure 4:**
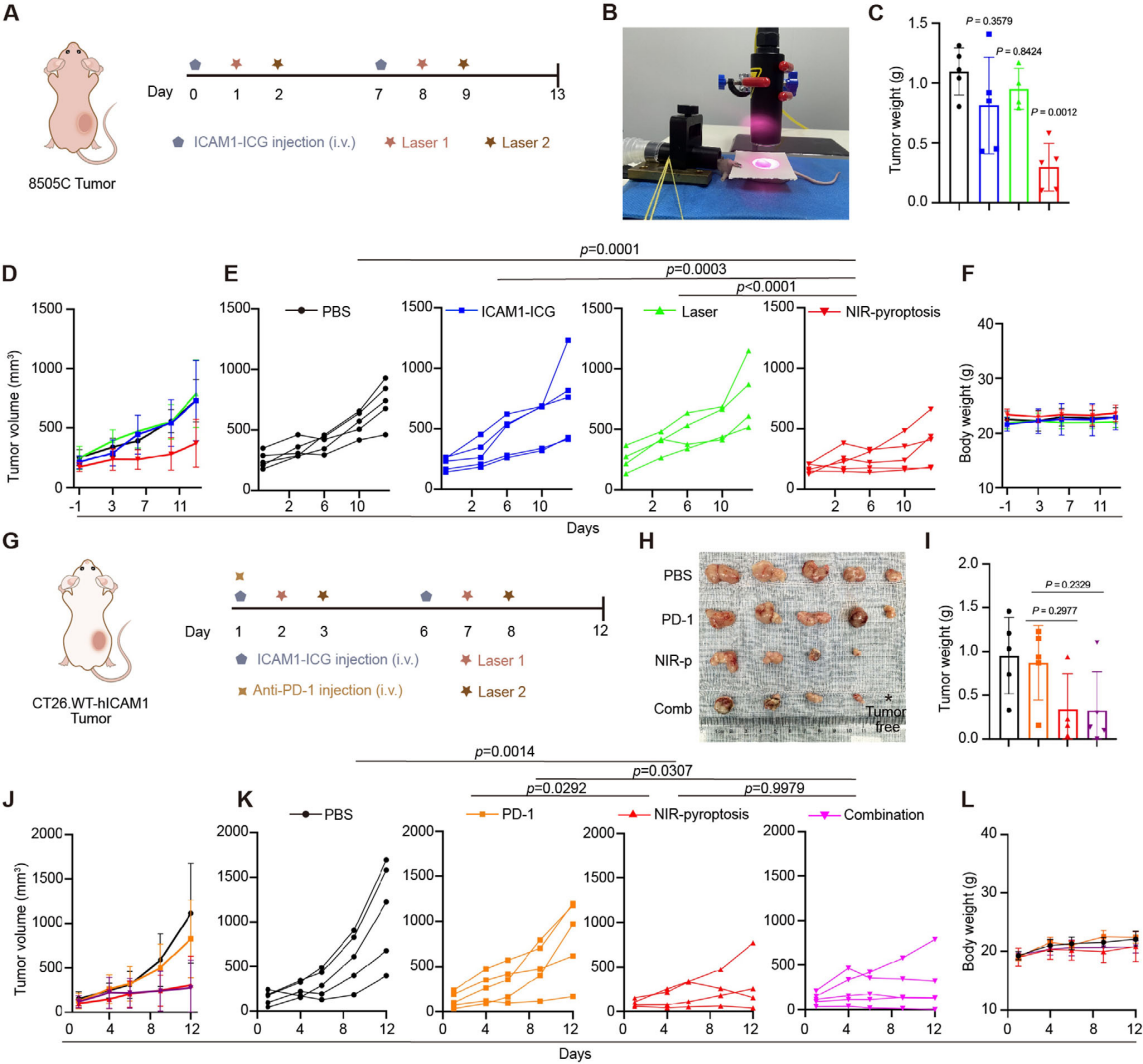
Antitumor effect of ICAM1‐targeted NIR‐pyroptosis in vivo. A) Schematic design of in vivo efficacy for NIR‐pyroptosis in an ATC tumor (8505C) xenografted nude mouse model. Laser1: 1 W cm^−2^, 64 s; Laser2: 1 W cm^−2^, 128 s. B) Image of NIR‐pyroptosis therapy in ATC tumor‐bearing nude mice. C) Tumor weight (at day 13) of mice in the ATC tumor xenografted nude mouse model. D) Tumor progression in the ATC tumor xenografted nude mouse model treated with PBS (n=5), ICAM1‐ICG (n=5), Laser (n=4), or NIR‐pyroptosis (n=5), respectively, and monitored by tumor volume measurement. E) Individual ATC tumor growth curves. F) Mouse body weight in the ATC tumor xenografted model. G) Schematic design of in vivo efficacy for NIR‐pyroptosis in a CT26.WT‐hICAM1 Balb/c mouse model. H) Image of excised CT26.WT‐hICAM1 tumors from mice treated with PBS (n=5), PD‐1 (n=5), NIR‐pyroptosis (n=4) or combination (n=5). I) Tumor weight (at day 12) of mice in the CT26.WT‐hICAM1 model. J) Tumor progression in the CT26.WT‐hICAM1 mouse model treated with PBS (n=5), PD‐1 (n=5), NIR‐pyroptosis (n=4) or combination (n=5), respectively, and monitored by tumor volume measurement. K) Individual CT26.WT‐hICAM1 tumor growth curves. L) Mouse bodyweight in the CT26.WT‐hICAM1 mouse model. In C) and I), data are presented as mean ± SD, and differences between each group are determined by One‐way ANOVA. In D) and J) data are presented as mean ± SD. The differences between each group are determined by Two‐way ANOVA.

To fully understand the therapeutic efficacy of NIR light‐triggered pyroptosis in vivo, we utilized a series of tumor models in both immunodeficient and immunocompetent tumor microenvironments. We first determined the in vivo efficacy of ICAM1‐ICG‐mediated NIR‐pyroptosis in a subcutaneous ATC tumor model (8505C) (**Figure** **4**A). Once the tumor volume reached ≈200 mm^3^, intravenous injection of 100 µg ICAM1‐ICG was administered. After 24 h, the tumor was locally irradiated with 1 W cm^−2^ of 808 nm NIR laser for 64 s, followed by a second irradiation of 1W cm^−2^ for 128 s after 48 h. The control groups were treated with equivalent doses of PBS, ICAM1‐ICG, or NIR laser irradiation, respectively. The same treatment regimen was repeated on the 7th day (Figure 4A,B). In a 14‐day course, the 8505C tumor growth of the ICAM1‐targeted NIR‐pyroptosis groups was potently attenuated in comparison with the control groups (Figure 4D,E). The NIR‐pyroptosis group exhibited a 73% reduction in tumor weight compared to the PBS group (Figure 4C). In comparison, ICAM1‐ICG or NIR laser irradiation alone showed only minimal impact on 8505C tumor progression.

Given that local pyroptosis typically induces immunogenic cell death (ICD) and promotes antitumor inflammatory responses,^[^ ^7^, ^45^ ^]^ we asked if ICAM1‐targeted NIR‐pyroptosis would work in synergy with PD‐1 immune checkpoint blockade (ICB) to further boost antitumor responses. To answer this question, we investigated the in vivo efficacy of ICAM1‐ICG in combination with systemic PD‐1 antibody treatment in an immunocompetent murine tumor model. An immunocompetent Balb/c mouse model was established by using murine colorectal carcinoma CT26 cells stably expressing human ICAM1 (CT26.WT‐hICAM1) owing to the unavailability of mouse ATC cell lines. ICAM1‐ICG or mouse PD‐1 monoclonal antibody was then intravenously administered at the same dosage of 100 µg, and NIR laser irradiation was consistent with the previous treatment regimen (Figure 4G). Our results demonstrated that NIR‐pyroptosis monotherapy resulted in a tumor growth inhibition of ≈64%, while the addition of anti‐PD‐1 modestly increased this to 66% (Figure 4I). This difference was not statistically significant. Since NIR‐pyroptosis alone has potently flattened the tumor growth curve, the combination group (NIR‐pyroptosis + PD‐1 antibody) did not further shrink tumor sizes (Figure 4H,J,K). The average tumor weights in different groups also support these conclusions (Figure 4I). Similarly, no body weight loss was observed in both nude mice and Balb/c mice (Figure 4F,L). Taken together, these results indicate that ICAM1‐ICG‐mediated NIR‐pyroptosis exhibits outstanding antitumor efficacy in both immunocompromised and immunocompetent models in vivo.

### 2.6 NIR‐Pyroptosis Therapy Boosts Anti‐Tumor Immunity

**Figure 5:**
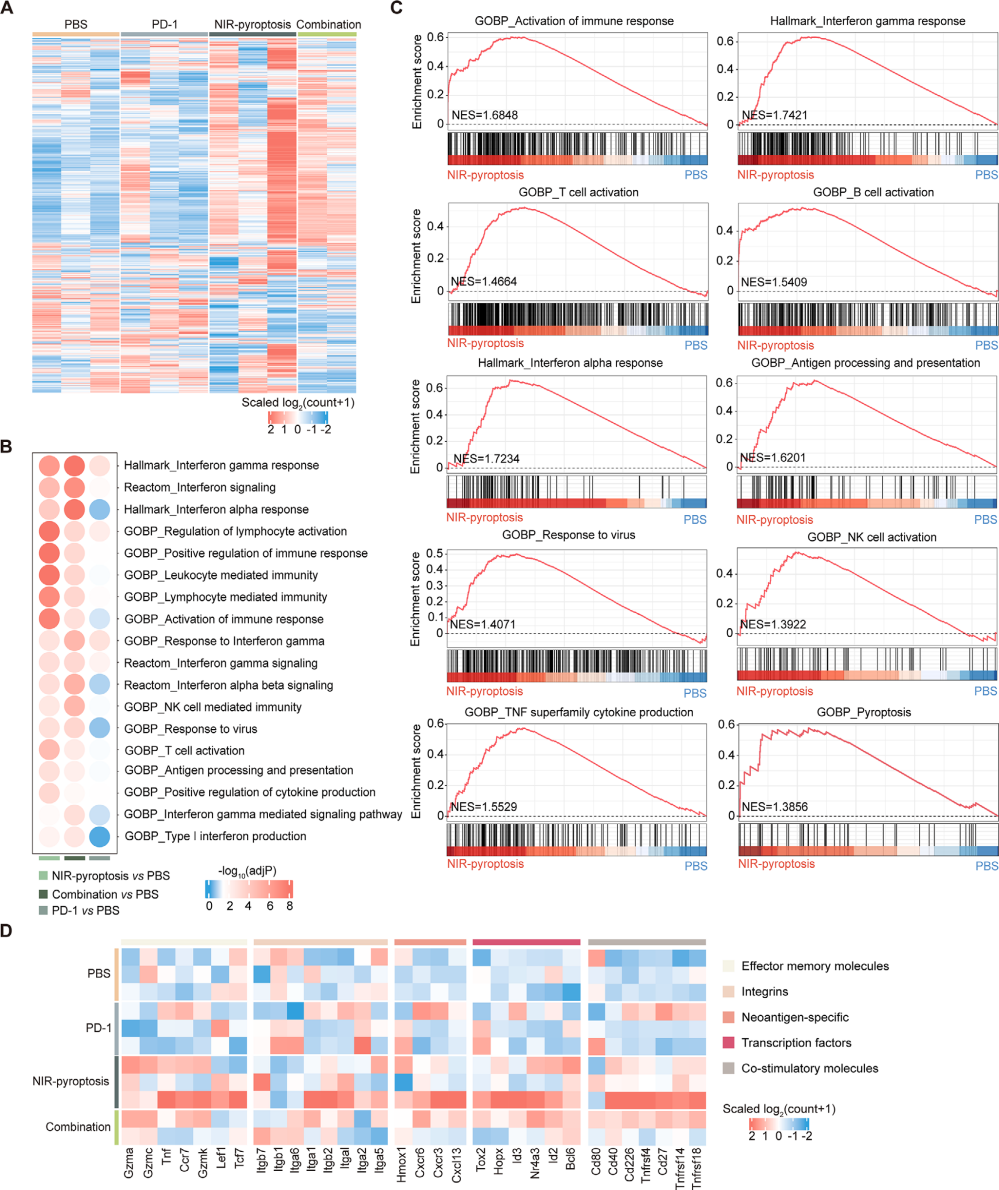
RNA‐seq analysis of CT26.WT‐hICAM1 tumors. A) Heatmap of top 1000 genes with significant expression in CT26.WT‐hICAM1 tumors treated with PBS (n = 3), PD‐1 (n = 3), NIR‐pyroptosis (n = 3), and combination (n = 2), respectively, and analyzed by RNA‐seq. B) Enrichment analysis of DEGs in CT26.WT‐hICAM1 tumors of Balb/c mice in each treatment group. C) GSEA of CT26.WT‐hICAM1 tumors, comparing NIR‐pyroptosis and PBS groups. D) Expression levels of selected marker genes in CT26.WT‐hICAM1 tumors of each indicated group.

To gain insight into the impact of NIR‐pyroptosis therapy on antitumor immunity, RNA‐seq transcriptomic analysis was carried out in CT26.WT‐hICAM1 tumors to reveal the response to various treatments, including PBS, PD‐1, NIR‐pyroptosis, and combination (NIR‐pyroptosis + PD‐1). By screening DEGs, we noted significant changes in gene expression in the NIR‐pyroptosis and combination therapy groups compared to the control group (PBS), while PD‐1 monotherapy showed minimal changes (**Figure** **5**A). Remarkably, the patterns of gene expression changes remained consistent between the NIR‐pyroptosis therapy and combination therapy groups. Based on these DEGs, we performed Gene Set Enrichment Analysis (GSEA) on immune‐related pathways, comparing each of the three treatment groups with the control group. The results demonstrated that both NIR‐pyroptosis therapy and combination therapy displayed congruent and substantial enrichment in immune‐related pathways, including the activation of immune response, interferon gamma response, interferon alpha response, and response to virus. In contrast, PD‐1 monotherapy exhibited limited enrichment in these pathways (Figure 5B,C). Meanwhile, enrichment of the pyroptosis pathway in tumor tissues echoed the cellular‐level results (Figures 5C and 2G). The consistency observed between the GSEA enrichment trend and the heatmap provides supporting evidence for our initial hypothesis that NIR‐triggered pyroptosis therapy and combination therapy have the potential to elicit antitumor immune responses. Moreover, CT26.WT‐hICAM1 tumors were enriched with many effector memory molecules, integrins, neoantigen‐specific markers, functional transcription factors, and costimulatory molecules after combination therapy or NIR‐triggered pyroptosis monotherapy (Figure 5D). These components have pivotal roles in prognosticating the effectiveness of cancer immunotherapy, as demonstrated in pertinent literature.^[^ ^46^ ^]^ Altogether, these RNA‐seq data showed that NIR‐pyroptosis therapy enhanced antitumor immune activity, thus giving a mechanistic explanation for their high efficacy.

### 2.7 NIR‐Pyroptosis Therapy is Generally Applicable to Other Targets

**Figure 6:**
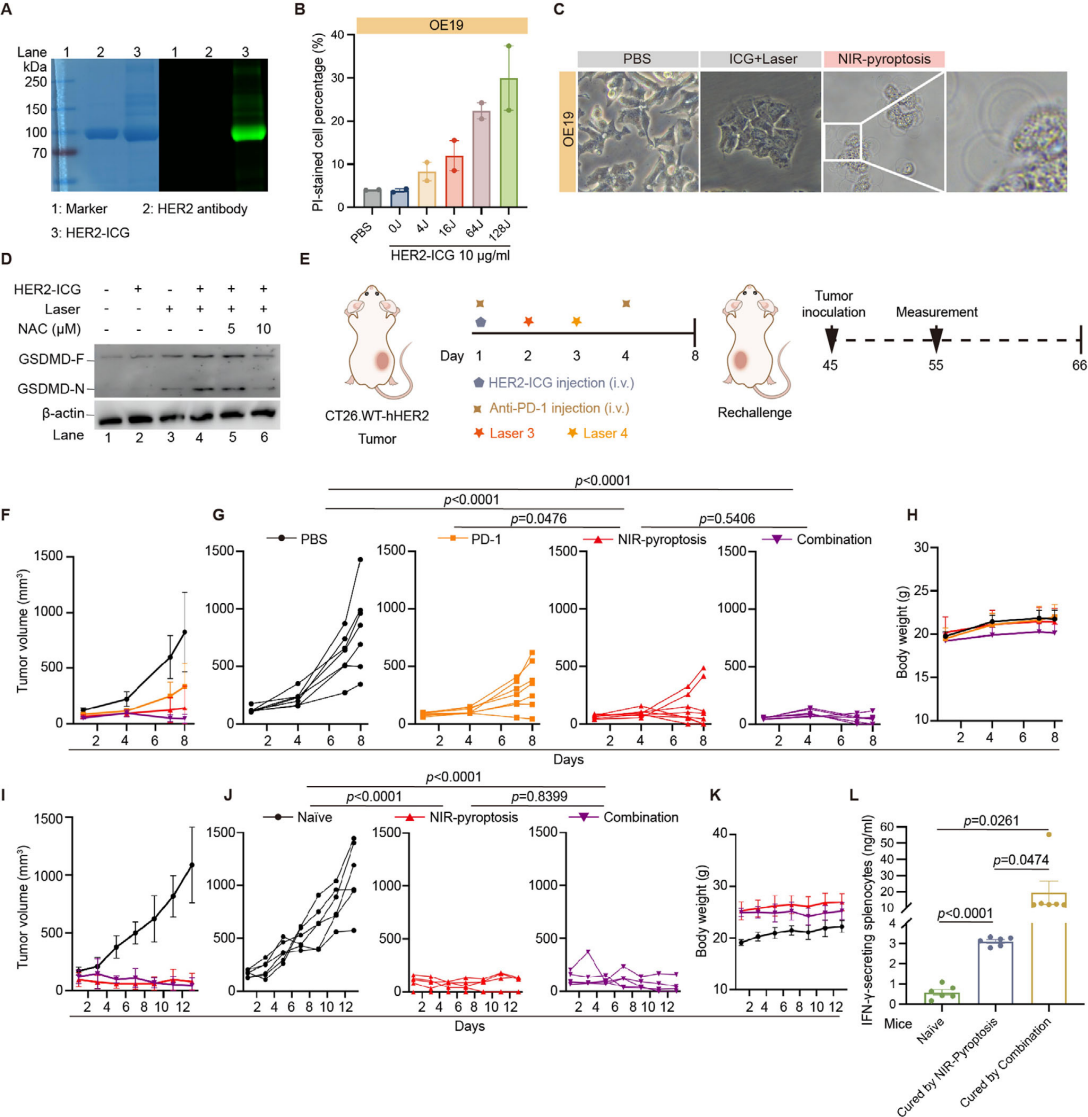
HER2‐targeted NIR‐pyroptosis induces antitumor effect and immunologic memory. A) Validation of HER2‐ICG by SDS‐PAGE (left: Colloidal Blue staining, right: fluorescence). Diluted HER2 mAb was used as a control. B) The in vitro cytotoxicity of HER2‐ICG on OE19 cells with a serial dose of NIR light irradiation using propidium iodide (PI) staining. C) Representative phase‐contrast cell images of OE19 cells treated with NIR‐pyroptosis. Arrows mark the cells showing pyroptotic morphology. D) Western blot analysis of GSDMD cleavage of OE19 cells with different treatments. GSDMD‐F, full‐length GSDMD; GSDMD‐N, N‐terminal cleavage product of GSDMD. E) Schematic design of in vivo efficacy for HER2‐targeted NIR‐pyroptosis in a CT26.WT‐hHER2 Balb/c mouse model, and the schematic design of the CT26.WT‐hHER2 tumor rechallenge model. Laser3: 1 W cm^−2^, 180 s; Laser4: 1 W cm^−2^, 300 s. F) Tumor progression in the CT26.WT‐hHER2 mouse model treated with PBS (n=7), PD‐1 (n=7), NIR‐pyroptosis (n=8) or combination (n=7), respectively, and monitored by tumor volume measurement. G) Individual CT26.WT‐ hHER2 tumor growth curves. H) Mouse body weight in the CT26.WT‐hHER2 model. I) Tumor progression in cured mice rechallenged with CT26.WT‐hHER2 tumor and monitored by tumor volume measurement. J) Individual CT26.WT‐hHER2 tumor growth curves. K) Mouse body weight in the rechallenge model. L) IFN‐γ secretion from splenocytes co‐cultured with CT26.WT‐hHER2 cells, as detected by ELISA. In L), data are presented as mean ± SD, and differences between each group are determined by One‐way ANOVA. In F) and I), data are presented as mean ± SD. The differences between each group are determined by Two‐way ANOVA.

Next, we investigated the potential universality of NIR‐pyroptosis therapy applied to other molecular targets independent of ICAM1. HER2 is the most commonly used ADC target in the clinic. Accordingly, we constructed an HER2‐targeted NIR‐pyroptosis ADC (HER2‐ICG, **Figures** **6**A and S1B, Supporting Information) by replacing ICAM1 antibody with trastuzumab, a clinically‐approved humanized HER2 monoclonal antibody. We then validated the in vitro cytotoxicity experiments and found that HER2‐ICG could also effectively facilitate NIR‐pyroptosis in HER2‐overexpressing human oesophageal carcinoma OE19 cells (Figure 6B–D).

Furthermore, an immunocompetent Balb/c mouse model was established by using murine colorectal carcinoma CT26 cells stably expressing human HER2 (CT26.WT‐hHER2) to investigate the in vivo efficacy of HER2‐ICG in combination with systemic PD‐1 antibodies (Figure 6E). Again, our results showed that HER2‐ICG‐mediated NIR‐pyroptosis, as well as the combination with PD‐1, effectively suppressed the growth of CT26.WT‐hHER2 tumors during the 8‐day treatment (Figure 6F,G). Notably, tumor eradication was seen in 62.5% (5/8) of CT26.WT‐hHER2‐bearing mice treated with NIR‐pyroptosis and 71.4% (5/7) treated with combined NIR‐pyroptosis and PD‐1 antibody without tumor recurrence for up to 38 days.

Next, these cured mice were rechallenged with CT26.WT‐hHER2 tumors (s.c. inoculation) in the opposite flank on day 45 (Figure 6E). Naïve mice, not previously inoculated with tumor cells, were used as a control group. Tumor volume and body weight were monitored starting from day 55. CT26.WT‐hHER2 tumors were completely suppressed in both rechallenged groups at rejection rates of 100% (5/5) in the NIR‐pyroptosis cured group and 100% (5/5) in the combined NIR‐pyroptosis and PD‐1 antibody cured group, respectively, whereas tumors grew normally in naïve mice (0/6, 0%) (Figure 6I,J).

This finding was also supported by the upregulation of IFN‐γ level detected in splenocytes. IFN‐γ is a cytokine primarily produced by activated T cells and natural killer (NK) cells, and it has antitumor effects.^[^ ^47^ ^]^ By the end of the rechallenge study, we isolated splenocytes from each mouse. Splenocytes from naïve mice challenged with CT26.WT‐HER2 (without NIR‐pyroptosis) showed limited response in terms of IFN‐γ secretion in response to CT26.WT‐hHER2 cells. In comparison, splenocytes from mice previously cured by NIR‐pyroptosis, or the combined treatment, and then rechallenged with CT26.WT‐hHER2 exhibited a significant activation response (Figure 6L). Similarly, no body weight loss was observed in any of the groups (Figure 6H,K). These data demonstrate that HER2‐ICG‐mediated NIR‐pyroptosis induced tumor‐specific immunologic memory, resulting in the recognition of human HER2 antigens by the immune system in mice previously inoculated with, but cured of, CT26.WT‐hHER2 tumors. These results gave evidence that NIR‐pyroptosis therapy could be applied to other molecular targets independent of ICAM1.

**Figure 7:**
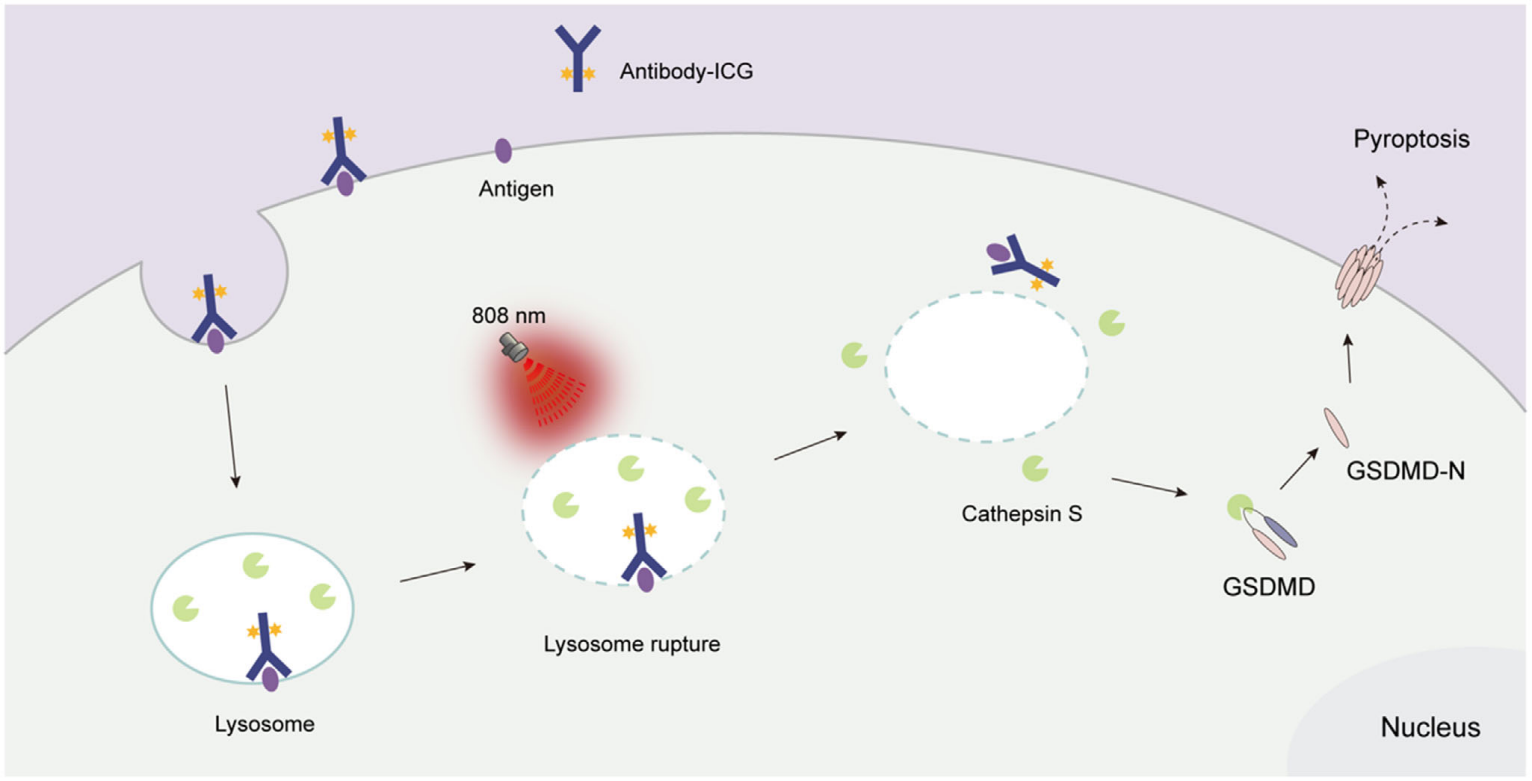
Schematic illustration of the non‐canonical pyroptosis mechanism via NIR‐light activated antibody‐ICG conjugates.

## 3 Discussion

Pyroptosis is a pivotal proinflammatory and non‐apoptotic PCD mechanism that holds immense potential for the development of next‐generation targeted therapeutics for solid tumors. In this study, we demonstrated that ICG, a clinically approved and safe fluorescent dye, can serve as a novel ADC warhead to induce NIR‐pyroptosis through a previously unrecognized biomechanism (Figure 7). Specifically, we identified that high concentrations of localized ROS in lysosomes, generated by NIR‐irradiated antibody‐bound ICG, induce lysosomal membrane damage, releasing lysosomal proteases into the cytosol. Among these, CTSS was uniquely identified as the lysosomal protease capable of cleaving GSDMD, IL‐1β, and IL‐18 independently of caspase‐1, establishing a novel pathway of tumor‐specific pyroptosis induction.

CTSS stands out as a cysteine protease due to its biochemical ability to remain enzymatically active at neutral pH, unlike most other cathepsin family members.^[^ ^33^ ^]^ This characteristic enhances its potential involvement in extra‐lysosomal proteolytic activities, such as MHC Class II antigen presentation via cleavage of the invariant chain.^[^ ^34^ ^]^ In our study, CTSS was identified as the primary driver of GSDMD‐N formation, an essential step in pyroptosis. GSDMD‐N oligomerization at the plasma membrane forms pores that facilitate the release of proinflammatory cytokines IL‐1β and IL‐18, while water influx results in cell swelling and lysis, hallmarks of pyroptotic cell death. To the best of our knowledge, this is the first report implicating CTSS in the direct cleavage of these proteins, bypassing the canonical caspase‐1‐dependent pathway.

This study also provides the first experimental evidence that ICG, a clinically approved and safe NIR dye, functions as a potent pyroptotic inducer when conjugated with antibodies. Previous approaches^[^ ^48^, ^49^, ^50^ ^]^using nanoparticulate systems to deliver free ICG for apoptotic cell death in photodynamic (PDT) or photothermal therapy (PTT) faced challenges such as high hepatic accumulation and poor tumor penetration due to reticuloendothelial system uptake.^[^ ^51^ ^]^ In contrast, antibodies offer distinct advantages as ICG carriers, including high tumor specificity, enhanced penetration, and a reduced molecular size (≈5 nm). Antibody‐bound ICG efficiently enters cancer cells through antigen‐mediated endocytosis and localizes within lysosomes, as evidenced by rapid uptake within 1 h (Figure 1H). This spatial specificity allowed ROS generated under NIR irradiation to induce localized lysosomal damage, a prerequisite for CTSS release into the cytosol. Unlike apoptosis, a non‐inflammatory form of PCD previously associated with ICG‐based photodynamic or photothermal therapies, the antibody‐ICG construct induced pyroptosis, as evidenced by balloon‐like vesicles, elevated IL‐1β/IL‐18 levels, and activation of GSDMD cleavage pathways. Moreover, ADC constructs, including antibody‐bound ICG, have demonstrated favorable safety profiles in humans and scalable manufacturing potential for clinical applications^16^, supported by the clinical success of cetuximab sarotalocan, the first NIR‐PIT ADC.

PCD, including apoptosis, necrosis, ferroptosis, pyroptosis, and autophagy, acts as a central mechanism for many cancer treatment modalities.^[^ ^52^, ^53^, ^54^, ^55^ ^]^ Pyroptosis is a proinflammatory PCD characterized by a unique phenotype of cell swelling associated with the influx of proinflammatory cytokines (IL‐1β and IL‐18), which is closely associated with malignant tumors, inflammatory diseases, and infectious diseases.^[^ ^56^, ^57^ ^]^ Classic pyroptosis is triggered by bacterial infections or innate immune stimuli, involving inflammasome activation that recruits pro‐caspase‐1 and other adaptor proteins. Pro‐caspase‐1 is activated through self‐cleavage, generating catalytically active caspase‐1. This enzyme cleaves pro‐IL‐1β and GSDMD, releasing IL‐1β and forming N‐terminal domain (GSDMD‐N)‐mediated membrane pores. These pores enable cytokine release and water influx, causing cell swelling, membrane rupture, and ultimately leading to cell pyroptosis.^[^ ^37^ ^]^

In contrast to this classical pathway, our study unveils a non‐canonical, caspase‐1‐independent mechanism mediated by CTSS, which uniquely cleaves GSDMD and IL‐1β directly, bypassing the need for caspase‐1. This mechanism is distinct from apoptosis, which has been commonly associated with ICG‐based photodynamic (PDT) or photothermal therapy (PTT).^[^ ^48^, ^49^, ^50^ ^]^ While prior studies linked ICG treatment to non‐inflammatory apoptotic death, our data show that cells treated with antibody‐bound ICG under NIR irradiation exhibit balloon‐like vesicles—a hallmark of pyroptosis.^[^ ^58^, ^59^ ^]^ These observations were corroborated by immunoblot and RNA‐seq analyses, which confirmed the activation of pyroptosis signaling pathways.

Furthermore, in experiments using ICAM1+ or HER2+ tumor cells treated with ICAM1‐ICG or HER2‐ICG constructs, NIR irradiation consistently induced pyroptosis. The tumor cells underwent lysosomal damage and pyroptotic death in a manner dependent on the antibody‐ICG construct, rather than free ICG. These results establish a direct and generally applicable link between NIR‐triggered pyroptosis and the tumor‐ablative effects of antibody‐bound ICG.

Finally, we evaluated the synergistic effects of NIR‐pyroptosis in combination with PD‐1 checkpoint blockade immunotherapy on tumor growth in vivo. Increasing evidence suggests that cancer cell pyroptosis can boost the host's antitumor immune activity, and when combined with immune checkpoint inhibitors, it has the potential to transform “cold tumors” into “hot tumors”, thereby exerting synergistic antitumor effects.^[^ ^7^ ^]^ Here, our results showed that both single NIR‐pyroptosis therapy and combination therapy halted CT26.WT‐hICAM1/hHER2 tumor growth compared to the mock control group. Complete regression of CT26.WT‐hHER2 tumors was achieved by prolonging exposure to NIR light irradiation and supplementing PD‐1 blockade post‐NIR. The complete eradication rates for single NIR‐pyroptosis therapy and combination therapy were 62.5% and 71.4%, respectively. Furthermore, immunocompetent mice cured by single NIR‐pyroptosis therapy and combination therapy completely rejected rechallenged CT26.WT‐hHER2 tumors with a rejection rate of 100%. Although our study did not comprehensively analyze the impact of NIR‐pyroptosis on innate and adaptive immune cell populations within the tumor, we did, indeed, observe a substantial increase in IFN‐γ levels in the supernatant of mouse splenocytes from NIR‐pyroptosis cured mice rechallenged with CT26.WT‐hHER2 cells. IFN‐γ is known to possess potent T cell and NK cell activating properties and augment their cytotoxicity against malignant cells.^[^ ^60^ ^]^ Additionally, RNA‐seq results from tumor tissues also support that NIR‐pyroptosis improves the system's adaptive immune response. All data illustrated the impressive immune activation capacity of NIR‐pyroptosis.

Despite the promising therapeutic potential of NIR‐pyroptosis demonstrated in this study, several limitations should be acknowledged. First, the limited tissue penetration depth of NIR light, typically restricted to 5–10 mm, presents a practical challenge for treating deep‐seated tumors. Consequently, our approach is best suited for accessible malignancies, such as head and neck cancers, cutaneous melanomas, or surgically exposed tumors, where direct irradiation is feasible. The clinical success of cetuximab sarotalocan (Akalux), the first photoactivatable payload‐containing ADC approved for NIR‐PIT,^[^ ^61^ ^]^ supports this concept. Akalux enables site‐specific NIR activation using either cylindrical diffusers inserted via needle catheters to treat deeper stromal tumors or frontal diffusers applied to superficial lesions. Second, although ICAM1 is significantly overexpressed in ATC and other aggressive cancers, its physiological expression in normal organs raises concerns about potential “on‐target, off‐tumor” toxicity. To mitigate this, our therapeutic platform is based on NIR‐pyroptosis, which employs spatially confined NIR light activation. Our preclinical in vivo data support this safety feature, with no systemic toxicity or body weight loss observed. Clinical precedents involving ICAM1‐targeted CAR‐T cells^[^ ^62^ ^]^ and oncolytic viruses^[^ ^63^ ^]^ have also demonstrated favorable safety, supporting the feasibility of ICAM1 as a therapeutic target, particularly when paired with tumor‐localized activation strategies such as NIR‐pyroptosis. Third, although the current NIR‐pyroptosis study did not include quantitative biodistribution experiments, we previously reported a thorough characterization of the antibody's in vivo behavior, showing ≈2.2‐fold higher tumor accumulation in an ATC xenograft model compared to non‐targeted controls, with minimal off‐target uptake in major organs, including the liver.^[^ ^64^ ^]^ Lastly, while both NIR‐pyroptosis monotherapy and its combination with PD‐1 blockade effectively suppressed tumor growth and induced durable immune memory, no statistically significant synergy was observed between the two. These additive rather than synergistic effects suggest that NIR‐pyroptosis alone may be sufficient to elicit a robust immunogenic response. Future studies should investigate alternative combinatorial strategies or optimized treatment regimens to further enhance immune synergy.

In summary, our research provides proof‐of‐concept evidence that ICG functions as a novel ADC warhead for NIR‐pyroptosis, driven by a previously unrecognized CTSS‐dependent mechanism. This innovative mechanism enables spatiotemporal induction of tumor cell pyroptosis while enhancing antitumor immunity. In immunocompetent animal models, this approach achieves potent and sustained tumor regression, with synergistic effects observed when combined with PD‐1 checkpoint immunotherapy, highlighting the broader potential of CTSS as a driver of pyroptosis in the development of next‐generation ADCs.

## 4 Experimental Section

### Bioinformatics Analysis of Cell Surface Protein Targets in ATC

Two ATC gene expression profile datasets, GSE65144 and GSE85457, were obtained from the GEO (https://www.ncbi.nlm.nih.gov/geo/) database. GSE65144 consists of 12 ATC and 13 normal thyroid samples, while GSE85457 comprises 4 ATC and 3 normal thyroid samples. Employing a threshold of P < 0.05 and |Log_2_(FC)| > 2, the GEO online tool GEO2R was used to identify differentially expressed genes (DEGs) between ATC and normal thyroid tissues. DEGs that were consistently upregulated in both datasets were defined as candidate genes. Next, the Human Protein Atlas database (HPA) (https://www.proteinatlas.org/) was used to investigate the subcellular localization of proteins encoded by these candidate genes, identifying four targets located on the cell membrane surface. The expression levels of these four targets in ATC cell lines (8505C) were compared utilizing the HPA database.

### Cell Culture, Plasmid Transfections, and Generation of Stable Cell Lines

The human ATC cell line 8505C was obtained from the Cell Culture Collection of the Chinese Academy of Sciences (Shanghai, China). Another human ATC cell line, TCO1, was obtained from the Japanese Collection of Research Bioresources (JCRB) Cell Bank. Human embryonic kidney 293T cells were obtained from the American Type Culture Collection (Manassas, VA, USA). Human carcinoma OE19 cells were purchased from Cobioer (Nanjing, China, Cat # CBP60495).

Mouse colorectal carcinoma CT26 cells stably expressing human ICAM1 (CT26.WT‐hICAM1), or stably expressing human HER2 (CT26.WT‐hHER2), were previously established by our laboratory. 8505C cells stably expressing human GSDMD‐EGFP were generated by infecting with GSDMD‐EGFP lentivirus (Genechem, Shanghai, China, Cat # GOSL0432449), following the manufacturer's protocol. Stably transduced cells were then selected with 2 µg mL^−1^ puromycin.

The pcDNA3.1‐CTSS‐3×Flag (Cat # G09015), pcDNA3.1‐GSDMD‐HA (Cat # C05008), and pcDNA3.1 vector were purchased from GenePharma (Shanghai, China). The cells were transfected with the indicated plasmids by lipofectamine 2000 (Invitrogen). The procedures described by the manufacturer were followed.

8505C, TCO1, and 293T cells were maintained in high‐glucose Dulbecco's modified Eagle's medium (DMEM, Gibco), and OE19 and CT26 were cultured in Roswell Park Memorial Institute (RPMI)‐1640 medium (Gibco). All cells were supplemented with 10% FBS (ExCell Bio) and 1% penicillin/streptomycin (Biosharp). All cells were maintained in a humidified atmosphere containing 5% CO_2_ at 37°C.

### Synthesis of NIR‐Pyroptosis ADC

Two different NIR‐pyroptosis ADCs were prepared via cysteine coupling. Briefly, purified anti‐human ICAM1 antibody (1 mg, mouse/human chimeric, GenScript) featuring a human IgG1 constant region (Fc) without cross‐reactivity to murine isoforms,^[^ ^65^ ^]^ was incubated with 5‐fold molar excess of ICG NHS ester (6.8 mg; Xarxbio, Cat # R‐TE‐157) in 0.1 mol L^−1^ Na_2_HPO_4_ (pH 8.5) at room temperature for 2 h. Trastuzumab (1 mg; Roche) was conjugated in parallel using the same procedure to generate a HER2‐targeted NIR‐pyroptosis ADC. The mixture was purified with a PD MiniTrap G‐25 column (Cytiva, Cat # 28918007) following the manufacturer's protocol. Protein concentration was determined with the BCA protein assay kit (Beyotime, Cat # P0012) by measuring the absorption at 562 nm with the Spark multimode microplate reader (TECAN). The average molecular weights of both unconjugated monoclonal antibodies and mAb‐ICG conjugates were determined using Matrix‐assisted laser desorption/ionization mass spectrometry (MALDI‐TOF‐MS) (***rapifleX*** ^®^ ***MALDI Tissuetyper*** ^®^, Brucker) with data analysis performed using flexAnalysis version 3.4. The Drug‐to‐Antibody Ratio (DAR) was calculated as DAR = (MW_mAb‐ICG_ ‐ MW_mAb_) / (MW_ICG‐NHS ester_ ‐115), where 115 represents the molecular mass of the N‐hydroxysuccinimide ester lost upon the coupling of ICG‐NHS ester to the monoclonalantibody.

As a quality control for the ADC conjugate, sodium dodecyl sulfate‐polyacrylamide gel electrophoresis (SDS‐PAGE) was performed. ICG‐conjugated ADC and unconjugated monoclonal antibody were separated by SDS‐PAGE with a 10% gradient polyacrylamide gel. After electrophoresis at 80 V for 2 h, the gel was imaged with an Amersham Typhoon (Cytiva) and then stained with Coomassie brilliant blue (Beyotime, Cat # P0017) to determine the molecular weight of the antibody‐ICG conjugate.

### Detection of Cell Surface ICAM1 Expression and its Internalization

For the detection of ATC cell surface Intercellular Adhesion Molecule 1 (ICAM1), also known as CD54 (Cluster of Differentiation 54), expression, three different methods were used, including immunofluorescence (IF) staining, (WB), and flow cytometry. For IF‐staining, 2.5 × 10^5^ cells were seeded into glass‐bottomed dishes. Upon reaching ≈80% confluence, the cells were fixed with 4% formaldehyde for 15 min at room temperature, followed by blocking with 1% BSA for 30 min on ice. Subsequently, they were stained with PE‐conjugated ICAM1 (BioLegend, Cat # 353106) or PE‐conjugated IgG (BioLegend, Cat # 410708) for 1 h at 4°C. After three rinses with PBS, the cells were stained with DAPI (Beyotime, Cat # C1005) for 7 min at room temperature. The fluorescence of the cells was observed using a confocal microscope (Nikon, A1 HD25).

For WB, whole‐cell lysates were prepared using lysis buffer (Beyotime, Cat # P0013) at 4°C for 30 min. Protein extract was then separated on a 10% polyacrylamide SDS gel and transferred to PVDF (Millipore) membranes and blocked with 5% milk for 2 h. The membrane was then immunoblotted with anti‐ICAM1 antibody (Sigma‐Aldrich, Cat # HPA002126) and anti‐beta actin (Abcam, Cat # ab8227) at 4 °C overnight. After washing with PBST, membranes were further incubated with HRP‐conjugated goat anti‐rabbit IgG (Abclonal, Cat # AS014) or anti‐mouse IgG (whole molecule)‐peroxidase antibody (Sigma, Cat # A9044) at room temperature for 2 h. Bands were visualized using a Western blot imaging system (Cytiva, Amersham ImageQuant™ 800).

For flow cytometry, a total of 1 × 10^6^ cells were harvested and rinsed twice with PBS and then blocked with 1% BSA for 30 min in an ice bath. Following BSA blocking, cells were incubated with PE‐conjugated ICAM1 or PE‐conjugated IgG antibodies for 1 h at room temperature, respectively. All samples were rinsed three times with PBS and determined in a CytoFLEX LX (Beckman Coulter) using FlowJo software (BD Biosciences).

To visualize the internalization of ICAM1 antibody on ATC cells, 2.5 × 10^5^ 8505C cells were seeded into glass‐bottomed dishes. When cells reached ≈80% confluence, they were rinsed twice with PBS, followed by a 30‐min incubation in 1% BSA at 4°C to block nonspecific binding. After blocking, the cells were co‐stained with PE‐conjugated ICAM1 for 1 h at 4°C, followed by three rinses with PBS. Subsequently, the samples were incubated at 37°C for 0, 30, 60, and 240 min. All samples were fixed with 4% paraformaldehyde for 15 min and then observed using a confocal microscope (Nikon, A1 HD25).

### In Vitro Efficacy Tests

For in vitro cytotoxicity of NIR‐pyroptosis, ATC cells were seeded in wells of a 12‐well plate at a density of 2.5×10^5^ cells per well. When the cells reached ≈80% confluence, the culture medium was replaced with fresh medium containing 10 µg mL^−1^ ICAM1‐ICG or HER2‐ICG and incubated for 6 h at 37°C. After washing with PBS, serial NIR light irradiation (808 nm, 1 W cm^−2^) was applied. 1 h after NIR laser irradiation, the cell suspension was incubated with propidium iodide (PI, Solarbio, Cat # CA1020) for 30 min at room temperature. PI‐positive cells were analyzed using CytoFLEX LX and FlowJo software.

### Transmission Electron Microscope (TEM)

8505C cells treated with PBS, ICAM1‐ICG, NIR laser, and NIR‐pyroptosis were collected and fixed with 2.5% glutaraldehyde at 4 °C overnight. After washing three times with PBS, the cells were fixed in 1% osmium tetroxide (OsO_4_) in PBS for 2 h at room temperature. Cells were then dehydrated in a graded series of ethanol solutions for 15 min and permeated in varying proportions of acetone and epoxy resin overnight. Subsequently, samples were cut into ultra‐thin sections (50–70 nm) and stained with 2% uranium dioxyacetate and lead citrate. Finally, cell images were observed under a transmission electron microscope (JEM‐2100plus, JEOL, Japan).

### RNA‐Seq

According to the instructions of LC‐Bio Technologies Co., Ltd. (Hangzhou, China), total RNA extraction from 8505C cells and CT26‐WT‐hICAM1 tumor tissues treated with NIR‐pyroptosis was separated with TRIzol, isolated by chloroform, and extracted using ethanol, respectively. Subsequently, cDNA library construction, library purification, and transcriptome sequencing were conducted for in‐depth data analysis.

### Western Blotting

A total of 2.5 × 10^5^ cells were seeded in each well of a 12‐well plate, and when the cells reached ≈80% confluence, the culture medium was replaced with fresh medium containing 10 µg mL^−1^ antibody‐ICG, and they were further incubated for at least 6 h. Subsequently, the cells were rinsed twice with PBS and subjected to 808nm irradiation at 1 W cm^−^^2^ for 128 s. After replacing with complete culture media, the cells were incubated for an additional 6 h before cell collection. Protein extraction, SDS‐PAGE, and membrane transfer were performed as described previously. The membrane was then subjected to immunoblotting with antibodies against β‐actin (CST, Cat # 4967S), caspase‐1 (Abcam, Cat # ab179515; Adipogen, Cat # AG‐20B‐0048‐C100), and GSDMD‐N‐terminal (Affinity, Cat # DF12275) at 4 °C overnight. Following washing with PBST, the membranes were further incubated with HRP‐labeled secondary antibodies at room temperature for 2 h. Protein bands were visualized using a Western blot imaging system.

### Measurement of Intracellular ROS Levels

Intracellular ROS levels were assessed using 2',7'‐dichlorofluorescein‐diacetate (DCFH‐DA) (Sigma, Cat # 2044‐85‐1), a non‐fluorescent compound that can be readily oxidized by intracellular ROS to form fluorescent dichlorofluorescein (DCF), its principal component. Consequently, ROS levels were quantified. After 4 h of NIR‐pyroptosis, the cells were incubated with a basal culture medium containing 10 µm DCFH‐DA for 20 min at 37°C, rinsed three times with PBS, and subsequently observed under a confocal microscope (Nikon, A1 HD25).

### Immunofluorescence Microscopy

Cells were cultured in confocal dishes and fixed with 4% paraformaldehyde for 15 min, permeabilized with 0.1% Triton X‐100 for 10 min, and blocked with 5% BSA for 40 min. Subsequently, the cells were incubated with the indicated primary antibodies: anti‐LAMP1 (Abcam, Cat # ab25630), anti‐Gal‐3(HUABIO, Cat # ER1803‐82), anti‐cathepsin S (Proteintech, Cat # 27538‐1‐AP) overnight at 4°C in the dark, followed by incubation with the specified secondary antibody: FITC conjugated goat anti‐mouse IgG polyclonal antibody (HUABIO, Cat # HA1003), Cy5.5 conjugated goat anti‐rabbit IgG polyclonal antibody (Bioss, Cat # bs‐0295G‐Cy5.5) at room temperature for 2 h. Nuclei were stained with Hoechst 33342 (ThermoFisher, Cat # 62249) for 10 min. Samples were observed using a confocal microscope (Nikon, A1 HD25) and a high‐sensitivity structured illumination microscope (HiS‐SIM).

For the colocalization between lysosome probe (Beyotime, Cat # C1047S) and ICAM‐ICG, after NIR‐pyroptosis was observed using confocal‐FLIM‐STED microscopy (Leica, Stellaris 8).

### In Vitro GSDMD, IL‐18, and IL‐1β Cleavage by Recombinant Cathepsin S

Recombinant cathepsin A (Cat # HY‐P7745), B (Cat # HY‐P7993A), D (Cat # HY‐P7748), E (Cat # HY‐P7750), Z (Cat # HY‐P7758), and S (Cat # HY‐P7756) were purchased from MCE (Shanghai, China). Recombinant GSDMD (Cat # CSB‐EP009956HUc7), IL‐18 (Cat # CSB‐EP614514HUd7), and IL‐1β (Cat # CSB‐EP011614HUa0) were purchased from CUSABIO (Wuhan, China). 2 µg of recombinant GSDMD, IL‐18 or IL‐1β was incubated with 1 µg of recombinant cathepsin S in a 25 µL reaction buffer containing 50 mm HEPES (pH 7.5), 3 mm EDTA, 150 mm NaCl, 0.005% (vol/vol) Tween‐20, and 10 mm DTT. The reaction was incubated for 60 min at 37°C. Cleavage of GSDMD, IL‐18 or IL‐1β was examined by Coomassie blue staining of the reaction samples separated on the SDS–PAGE gel. The specific inhibitor of cathepsin S, LY 3000328 (Z‐FL‐COCHO, ZFL) (Cat # HY‐15533), was purchased from MCE.

### In Vivo Efficacy Tests

The animal experiments in this study were performed in accordance with the protocols approved by the Institutional Animal Care and Use Committee (IACUC) of the Hangzhou Institute of Medicine, Chinese Academy of Sciences. The in vivo efficacy of NIR‐pyroptosis was tested in three animal models, including an ATC tumor (8505C) xenografted nude mouse model, a CT26.WT‐hICAM1 tumor Balb/c mouse model, and a CT26.WT‐hHER2 tumor Balb/c mouse model. For the ATC tumor (8505C) model, 5 × 10^6^ 8505C cells, containing 50% Matrigel, were subcutaneously injected into the right flank of 4–6‐week‐old female nude mice. When the tumor volume reached ≈200 mm^3^, the mice were randomly divided into four groups (n ≥ 5) and subjected to PBS, ICAM1‐ICG, NIR irradiation, or NIR‐pyroptosis treatment. ICAM1‐ICG injection was administered at a dosage of 5 mg kg^−1^ per mouse. NIR irradiation was applied at a dose of 64 J cm^−2^ on day 1 and 128 J cm^−2^ on day 2. This treatment regimen was repeated once a week for a total duration of two weeks. The tumor volume was monitored three times per week and calculated as V = L × W^2^ × π/6. The endpoint was reached after two weeks of treatment, and mice were anesthetized with 2,2,2‐tribromoethanol and euthanized. Subcutaneous tumors were excised to measure the mass.

For the CT26.WT‐hICAM1 tumor model, 5 × 10^6^ CT26‐WT‐hICAM1 cells, mixed with 50% Matrigel, were subcutaneously injected into the right flank of 4–6‐week‐old female Balb/c mice. When the tumor volume reached ≈800‐1000 mm^3^, the mice were anesthetized with 2,2,2‐tribromoethanol and then euthanized. Subcutaneous tumors were excised, cut into 8 mm^3^ pieces, and then subcutaneously transplanted into the right dorsal flank of 4‐6‐week‐old female Balb/c mice. When the tumor volume reached ≈200 mm^3^, the mice were randomly divided into four groups (n ≥ 5) and subjected to PBS, PD‐1 antibody, NIR‐pyroptosis, or combined NIR‐pyroptosis and PD‐1 antibody treatment. ICAM1‐ICG injection and NIR irradiation followed the previously mentioned protocol, and PD‐1 antibody injection was administered at a dose of 5 mg kg^−1^ of body weight per mouse. Treatments were administered weekly for two weeks. At the study endpoint, mice were euthanized after anesthesia, and subcutaneous tumors were excised for mass measurement.

Applying the same protocol as described above, a CT26‐WT‐hHER2 subcutaneous tumor model was established in 4‐6‐week‐old female Balb/c mice. Injection of HER2‐ICG was consistent with the previous treatment with NIR irradiation doses of 180 J cm^−2^ (day 1) and 300 J cm^−2^ (day 2). In the combination group, PD‐1 antibodies were administered before and after NIR irradiation at the same dose of 5 mg kg^−1^ of body weight per mouse. Tumor volume was monitored for one week.

Starting with mice whose CT26.WT‐hHER2 tumors had been cured by NIR‐pyroptosis and combination therapy, the rechallenge study employed subcutaneous injection with 8 mm^3^ fragments of CT26.WT‐hHER2 tumors into the left flank. Another group of mice not previously tumor‐inoculated was also injected with CT26.WT‐hHER2 tumors for comparison. Tumor volume was monitored, starting at ≈100 mm^3^ and continuing for 12 days. At the endpoint, mice were euthanized after anesthesia, spleens were excised, and splenocytes were prepared to assess the response to CT26.WT‐hHER2 cells.

### ELISA

Splenocytes from CT26.WT‐hHER2 tumor‐bearing naïve mice without drug treatment and mice whose CT26.WT‐hHER2 tumors had been cured by NIR‐pyroptosis and combination therapy were subsequently co‐cultured with CT26.WT‐hHER2 cells at 37°C and 5% CO_2_ for 24 h in wells of a 96‐well plate. Subsequently, cell culture supernatants were collected, and the secretion of IFN‐γ in the co‐cultured cells was determined using a mouse IFN‐γ ELISA kit (MULTISCIENCES, Cat # EK280) according to the manufacturer's instructions.

### Statistical Analysis

All statistical analyses were performed using GraphPad Prism 9.0.0. Statistical significance is indicated in the figures. One‐way ANOVA and Two‐way ANOVA were used to compare the differences between each group. P<0.05 was considered statistically significant.

Additional details are described in the Supporting Information.

## Conflict of Interest

F.C., W.H.Z. and P.G. are co‐inventors of a patent filed by Zhejiang Cancer Hospital and Hangzhou Institute of Medicine, Chinese Academy of Sciences. X.F.L. and J.F. are shareholders of MabPlex. The other authors declare no conflict of interest.

## Author Contributions

F.C. and X.‐F. T. contributed equally to this work and are co‐first authors. Y.L., W.H.Z., P.G., W.H.T. contributed equally to this work and are co‐corresponding authors. P.G. and F.C. did the conceptualization. F.C., X.F.T., Y.T., Y.J.D., D.Y.C., H.B.C., Y.L., and CLS did the methodology. F.C. and X.F.T. did the Investigation. Y.J.D., T.S., X.F.L., Q.J., J.C., M.Y.F., J.B.S., J.M.F., and W.H.Z. did supervision. F.C. and P.G. wrote the original draft. F.C., P.G., and W.H.T. Review & Editing.

## Supporting information



Supporting Information

## Data Availability

The data that support the findings of this study are available in the supplementary material of this article.

## References

[1] F. Llambi , D. R. Green , Curr. Opin. Genet. Dev. 2011, 21, 12. 21236661 10.1016/j.gde.2010.12.001 PMC3040981

[2] A. R. Delbridge , S. Grabow , A. Strasser , D. L. Vaux , Nat. Rev. Cancer 2016, 16, 99. 26822577 10.1038/nrc.2015.17

[3] S. K. Hsu , C. Y. Li , I. L. Lin , W. J. Syue , Y. F. Chen , K. C. Cheng , Y. N. Teng , Y. H. Lin , C. H. Yen , C. C. Chiu , Theranostics 2021, 11, 8813. 34522213 10.7150/thno.62521 PMC8419056

[4] J. Yan , P. Wan , S. Choksi , Z. G. Liu , Trends Cancer 2022, 8, 21. 34627742 10.1016/j.trecan.2021.09.003 PMC8702466

[5] Y. Liu , Z. Song , Y. Liu , X. Ma , W. Wang , Y. Ke , Y. Xu , D. Yu , H. Liu , Acta Pharm. Sin. B 2021, 11, 1513. 34221865 10.1016/j.apsb.2021.05.006 PMC8245858

[6] W. Gao , X. Wang , Y. Zhou , X. Wang , Y. Yu , Signal Transduct Target Ther 2022, 7, 196. 35725836 10.1038/s41392-022-01046-3 PMC9208265

[7] Z. Zhang , Y. Zhang , S. Xia , Q. Kong , S. Li , X. Liu , C. Junqueira , K. F. Meza‐Sosa , T. M. Y. Mok , J. Ansara , S. Sengupta , Y. Yao , H. Wu , J. Lieberman , Nature 2020, 579, 415. 32188940 10.1038/s41586-020-2071-9 PMC7123794

[8] Q. Wang , Y. Wang , J. Ding , C. Wang , X. Zhou , W. Gao , H. Huang , F. Shao , Z. Liu , Nature 2020, 579, 421. 32188939 10.1038/s41586-020-2079-1

[9] Z. Zhang , Y. Zhou , S. Zhao , L. Ding , B. Chen , Y. Chen , Adv. Sci. (Weinh) 2022, 9, 2203583. 36266982 10.1002/advs.202203583 PMC9762308

[10] F. Yang , S. N. Bettadapura , M. S. Smeltzer , H. Zhu , S. Wang , Acta Pharmacol. Sin. 2022, 43, 2462. 35288674 10.1038/s41401-022-00887-6 PMC9525650

[11] Y. Zhang , Q. Jia , J. Li , J. Wang , K. Liang , X. Xue , T. Chen , L. Kong , H. Ren , W. Liu , P. Wang , J. Ge , Adv. Mater. 2023, 35, 2305073. 10.1002/adma.202305073 37421648

[12] C. H. Chau , P. S. Steeg , W. D. Figg , Lancet 2019, 394, 793. 31478503 10.1016/S0140-6736(19)31774-X

[13] Z. Fu , S. Li , S. Han , C. Shi , Y. Zhang , 2022, 7, 93. 10.1038/s41392-022-00947-7 PMC8941077 35318309

[14] Y. A. Heo , Drugs 2023, 83, 265. 36656533 10.1007/s40265-023-01834-3

[15] M. J. Birrer , K. N. Moore , I. Betella , R. C. Bates , J Natl Cancer Inst 2019, 111, 538. 30859213 10.1093/jnci/djz035

[16] N. L. Miyazaki , A. Furusawa , P. L. Choyke , H. Kobayashi , Cancers (Basel) 2023, 15, 5117. 37958293 10.3390/cancers15215117 PMC10650558

[17] M. Ogawa , Y. Tomita , Y. Nakamura , M. J. Lee , S. Lee , S. Tomita , T. Nagaya , K. Sato , T. Yamauchi , H. Iwai , A. Kumar , T. Haystead , H. Shroff , P. L. Choyke , J. B. Trepel , H. Kobayashi , Oncotarget 2017, 8, 10425. 28060726 10.18632/oncotarget.14425 PMC5354669

[18] K. Sato , K. Ando , S. Okuyama , S. Moriguchi , T. Ogura , S. Totoki , H. Hanaoka , T. Nagaya , R. Kokawa , H. Takakura , M. Nishimura , Y. Hasegawa , P. L. Choyke , M. Ogawa , H. Kobayashi , ACS Cent. Sci. 2018, 4, 1559. 30555909 10.1021/acscentsci.8b00565 PMC6276043

[19] M. A. Hsu , S. M. Okamura , C. D. De Magalhaes Filho , D. M. Bergeron , A. Rodriguez , M. West , D. Yadav , R. Heim , J. J. Fong , M. Garcia‐Guzman , Cancer Immunol. Immunother. 2023, 72, 151. 35776159 10.1007/s00262-022-03239-9 PMC9813181

[20] K. C. Bible , E. Kebebew , J. Brierley , J. P. Brito , M. E. Cabanillas , T. J. Clark , A. Di Cristofano , R. Foote , T. Giordano , J. Kasperbauer , K. Newbold , Y. E. Nikiforov , G. Randolph , M. S. Rosenthal , A. M. Sawka , M. Shah , A. Shaha , R. Smallridge , C. K. Wong‐Clark , Thyroid 2021, 31, 337. 33728999 10.1089/thy.2020.0944 PMC8349723

[21] M. Varanese , S. Arcieri , A. Lauro , C. Panetta , C. Eberspacher , R. Palma , D. Mascagni , S. Pontone , Surg Innov 2024, 31, 103. 37923725 10.1177/15533506231209127

[22] B. Pan , Y. Yuan , Z. Yang , D. Lu , T. Long , Y. Sun , S. Yin , F. Zhang , Gland Surg 2023, 12, 1276. 37842534 10.21037/gs-23-242 PMC10570973

[23] E. Cervera‐Taulet , J. Montero‐Hernandez , C. Monferrer Adsuara , J. S. Pulido , Eur. J. Ophthalmol. 2024, 34, NP46. 37649336 10.1177/11206721231199337

[24] Y. Liao , J. Zhao , Y. Chen , B. Zhao , Y. Fang , F. Wang , C. Wei , Y. Ma , H. Ji , D. Wang , D. Tang , Cancers (Basel) 2022, 14, 5143. 36291927 10.3390/cancers14205143 PMC9601265

[25] S. Niu , Y. Liu , D. Li , Y. Sheng , Y. Zhang , Z. Li , S. Zhao , T. Wang , Front. Oncol. 2023, 13, 1257585. 37766867 10.3389/fonc.2023.1257585 PMC10520705

[26] J. Bombardelli , S. Farhat , A. De La Fuente Hagopian , J. Hua , M. A. Schusterman , Plast Reconstr Surg Glob Open 2023, 11, 5230. 10.1097/GOX.0000000000005230 PMC10482082 37681066

[27] S. Takayama , K. Ishikawa , H. Kani , S. Takayama , M. Sakamoto , Cureus 2022, 14, 22184. 10.7759/cureus.22184 PMC8923255 35308765

[28] M. Caimano , G. Bianco , A. Coppola , G. Marrone , S. Agnes , Q. Lai , G. Spoletini , Int J Surg 2024, 110, 431. 37800567 10.1097/JS9.0000000000000779 PMC10793811

[29] P. Ott , Pharmacol Toxicol 1998, 83, 1. 10.1111/j.1600-0773.1998.tb01945.x 9695126

[30] I. Yoon , J. Z. Li , Y. K. Shim , Clin Endosc 2013, 46, 7. 23423543 10.5946/ce.2013.46.1.7 PMC3572355

[31] P. Agostinis , K. Berg , K. A. Cengel , T. H. Foster , A. W. Girotti , S. O. Gollnick , S. M. Hahn , M. R. Hamblin , A. Juzeniene , D. Kessel , M. Korbelik , J. Moan , P. Mroz , D. Nowis , J. Piette , B. C. Wilson , J. Golab , CA Cancer J Clin 2011, 61, 250. 21617154 10.3322/caac.20114 PMC3209659

[32] N. Desai , V. Trieu , Z. Yao , L. Louie , S. Ci , A. Yang , C. Tao , T. De , B. Beals , D. Dykes , P. Noker , R. Yao , E. Labao , M. Hawkins , P. Soon‐Shiong , Clin. Cancer Res. 2006, 12, 1317. 16489089 10.1158/1078-0432.CCR-05-1634

[33] H. A. Chapman , R. J. Riese , G. P. Shi , Annu. Rev. Physiol. 1997, 59, 63. 9074757 10.1146/annurev.physiol.59.1.63

[34] K. Honey , A. Y. Rudensky , Nat. Rev. Immunol. 2003, 3, 472. 12776207 10.1038/nri1110

[35] C. Egloff‐Juras , L. Bezdetnaya , G. Dolivet , H. P. Lassalle , Int J Nanomedicine 2019, 14, 7823. 31576126 10.2147/IJN.S207486 PMC6768149

[36] A. K. Kirchherr , A. Briel , K. Mader , Mol Pharm 2009, 6, 480. 19228053 10.1021/mp8001649

[37] J. Ding , K. Wang , W. Liu , Y. She , Q. Sun , J. Shi , H. Sun , D. C. Wang , F. Shao , Nature 2016, 535, 111. 27281216 10.1038/nature18590

[38] Y. Wang , W. Gao , X. Shi , J. Ding , W. Liu , H. He , K. Wang , F. Shao , Nature 2017, 547, 99. 28459430 10.1038/nature22393

[39] B. Chen , Y. Yan , Y. Yang , G. Cao , X. Wang , Y. Wang , F. Wan , Q. Yin , Z. Wang , Y. Li , L. Wang , B. Xu , F. You , Q. Zhang , Y. Wang , Nat. Nanotechnol. 2022, 17, 788. 35606443 10.1038/s41565-022-01125-0

[40] R. Radzi , T. Osaki , T. Tsuka , T. Imagawa , S. Minami , Y. Nakayama , Y. Okamoto , J Vet Med Sci 2012, 74, 545. 22146339 10.1292/jvms.11-0464

[41] C. Shirata , J. Kaneko , Y. Inagaki , T. Kokudo , M. Sato , S. Kiritani , N. Akamatsu , J. Arita , Y. Sakamoto , K. Hasegawa , N. Kokudo , Sci. Rep. 2017, 7, 13958. 29066756 10.1038/s41598-017-14401-0 PMC5654824

[42] C. P. Rosa , T. C. A. Belo , N. C. M. Santos , E. N. Silva , J. Gasparotto , P. P. Corsetti , L. A. de Almeida , Life Sci. 2023, 331, 122076. 37683723 10.1016/j.lfs.2023.122076

[43] I. Paz , M. Sachse , N. Dupont , J. Mounier , C. Cederfur , J. Enninga , H. Leffler , F. Poirier , M. C. Prevost , F. Lafont , P. Sansonetti , Cell. Microbiol. 2010, 12, 530. 19951367 10.1111/j.1462-5822.2009.01415.x

[44] I. Maejima , A. Takahashi , H. Omori , T. Kimura , Y. Takabatake , T. Saitoh , A. Yamamoto , M. Hamasaki , T. Noda , Y. Isaka , T. Yoshimori , EMBO J. 2013, 32, 2336. 23921551 10.1038/emboj.2013.171 PMC3770333

[45] Z. Zhang , Y. Zhang , J. Lieberman , Cancer Immunol. Res. 2021, 9, 2. 33397791 10.1158/2326-6066.CIR-20-0525 PMC7789047

[46] M. Zhang , Y. Huang , J. Pan , C. Sang , Y. Lin , L. Dong , X. Shen , Y. Wu , G. Song , S. Ji , F. Liu , M. Wang , Y. Zheng , S. Zhang , Z. Wang , J. Ren , D. Gao , J. Zhou , J. Fan , W. Wei , J. Lin , Q. Gao , Cancer Discov 2023, 13, 2248. 37486241 10.1158/2159-8290.CD-23-0282

[47] L. B. Ivashkiv , Nat. Rev. Immunol. 2018, 18, 545. 29921905 10.1038/s41577-018-0029-z PMC6340644

[48] Y. Lu , L. Chen , Z. Wu , P. Zhou , J. Dai , J. Li , Q. Wen , Y. Fan , F. Zeng , Y. Chen , S. Fu , Biomed. Pharmacother. 2023, 169, 115846. 37944443 10.1016/j.biopha.2023.115846

[49] W. Chen , M. Zhang , C. Wang , Q. Zhang , ACS Appl. Mater. Interfaces 2023, 15, 55433. 37976376 10.1021/acsami.3c13405

[50] J. Liu , Y. Yin , L. Yang , B. Lu , Z. Yang , W. Wang , R. Li , Int J Nanomedicine 2021, 16, 1473. 33654397 10.2147/IJN.S284518 PMC7910086

[51] E. Blanco , H. Shen , M. Ferrari , Nat. Biotechnol. 2015, 33, 941. 26348965 10.1038/nbt.3330 PMC4978509

[52] Y. Luo , W. Tang , S. Xiang , J. Feng , X. Zu , Cancer Lett 2022, 550, 215929. 36202173 10.1016/j.canlet.2022.215929

[53] M. Scimeca , V. Rovella , V. Palumbo , M. P. Scioli , R. Bonfiglio , C. Tor , G. Melino , M. Piacentini , L. Frati , M. Agostini , E. Candi , A. Mauriello , Cancers (Basel) 2023, 15, 3638. 37509299 10.3390/cancers15143638 PMC10377326

[54] G. J. Yoshida , J. Hematol. Oncol. 2017, 10, 67. 28279189 10.1186/s13045-017-0436-9 PMC5345270

[55] D. Qi , M. Peng , Front Immunol 2023, 14, 1188365. 37325669 10.3389/fimmu.2023.1188365 PMC10264078

[56] Z. Rao , Y. Zhu , P. Yang , Z. Chen , Y. Xia , C. Qiao , W. Liu , H. Deng , J. Li , P. Ning , Z. Wang , Theranostics 2022, 12, 4310. 35673561 10.7150/thno.71086 PMC9169370

[57] S. Verdonck , J. Nemegeer , P. Vandenabeele , J. Maelfait , Trends Microbiol. 2022, 30, 593. 34933805 10.1016/j.tim.2021.11.011

[58] T. Bergsbaken , S. L. Fink , B. T. Cookson , Nat. Rev. Microbiol. 2009, 7, 99. 19148178 10.1038/nrmicro2070 PMC2910423

[59] J. Shi , Y. Zhao , K. Wang , X. Shi , Y. Wang , H. Huang , Y. Zhuang , T. Cai , F. Wang , F. Shao , Nature 2015, 526, 660. 26375003 10.1038/nature15514

[60] C. Nakajima , Y. Uekusa , M. Iwasaki , N. Yamaguchi , T. Mukai , P. Gao , M. Tomura , S. Ono , T. Tsujimura , H. Fujiwara , T. Hamaoka , Cancer Res. 2001, 61, 3399. 11309299

[61] D. M. Cognetti , J. M. Johnson , J. M. Curry , S. T. Kochuparambil , D. McDonald , F. Mott , M. J. Fidler , K. Stenson , N. R. Vasan , M. A. Razaq , J. Campana , P. Ha , G. Mann , K. Ishida , M. Garcia‐Guzman , M. Biel , A. M. Gillenwater , Head Neck 2021, 43, 3875. 34626024 10.1002/hed.26885 PMC9293150

[62] S. A. Srour , V. M. Villaflor , J. H. Lorch , M. E. Zafereo , S. Gupta , M. I.‐N. Hu , R. Dadu , A. Y. Lin , Y. Lu , L. Ackatz , M. Cushing , S. Avecilla , T. Scognamiglio , M. Jin , J. Puc , G. Liu , K. Du , S. A. Mayer , K. V. Besien , M. E. Cabanillas , J. Clin. Oncol. 2024, 42, 6112.

[63] N. E. Annels , D. Mansfield , M. Arif , C. Ballesteros‐Merino , G. R. Simpson , M. Denyer , S. S. Sandhu , A. A. Melcher , K. J. Harrington , B. Davies , G. Au , M. Grose , I. Bagwan , B. Fox , R. Vile , H. Mostafid , D. Shafren , H. S. Pandha , Clin. Cancer Res. 2019, 25, 5818. 31273010 10.1158/1078-0432.CCR-18-4022

[64] P. Zhang , C. Tao , T. Shimura , A. C. Huang , N. Kong , Y. Dai , S. Yao , Y. Xi , X. Wang , J. Fang , M. A. Moses , P. Guo , iScience 2023, 26, 107272. 37520726 10.1016/j.isci.2023.107272 PMC10371847

[65] P. Guo , J. Huang , B. Zhu , A. C. Huang , L. Jiang , J. Fang , M. A. Moses , Sci. Adv. 2023, 9, abq7866. 10.1126/sciadv.abq7866 PMC10162665 37146146

