# Supplementary material for: Lysosomal Cathepsin S Escape Facilitates Near Infrared Light‐Triggered Pyroptosis Via an Antibody‐Indocyanine Green Conjugate
Source: Adv Sci (Weinh). 2025 Jun 20;12(34):e04851. doi: 10.1002/advs.202504851 (PMC12442674; doi:10.1002/advs.202504851)
Supplement: Supplementary file 1 — Supporting Information [file ADVS-12-e04851-s001.docx]

**Lysosomal cathepsin S escape facilitates near infrared light-triggered pyroptosis via an antibody-indocyanine green conjugate**

**Supplement**

**
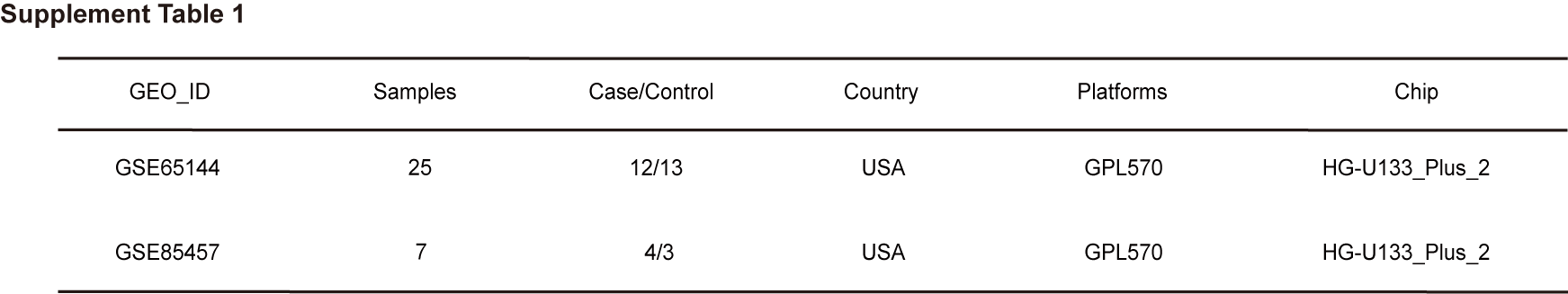
**

**Table S1.** ATC dataset in GEO database.

**
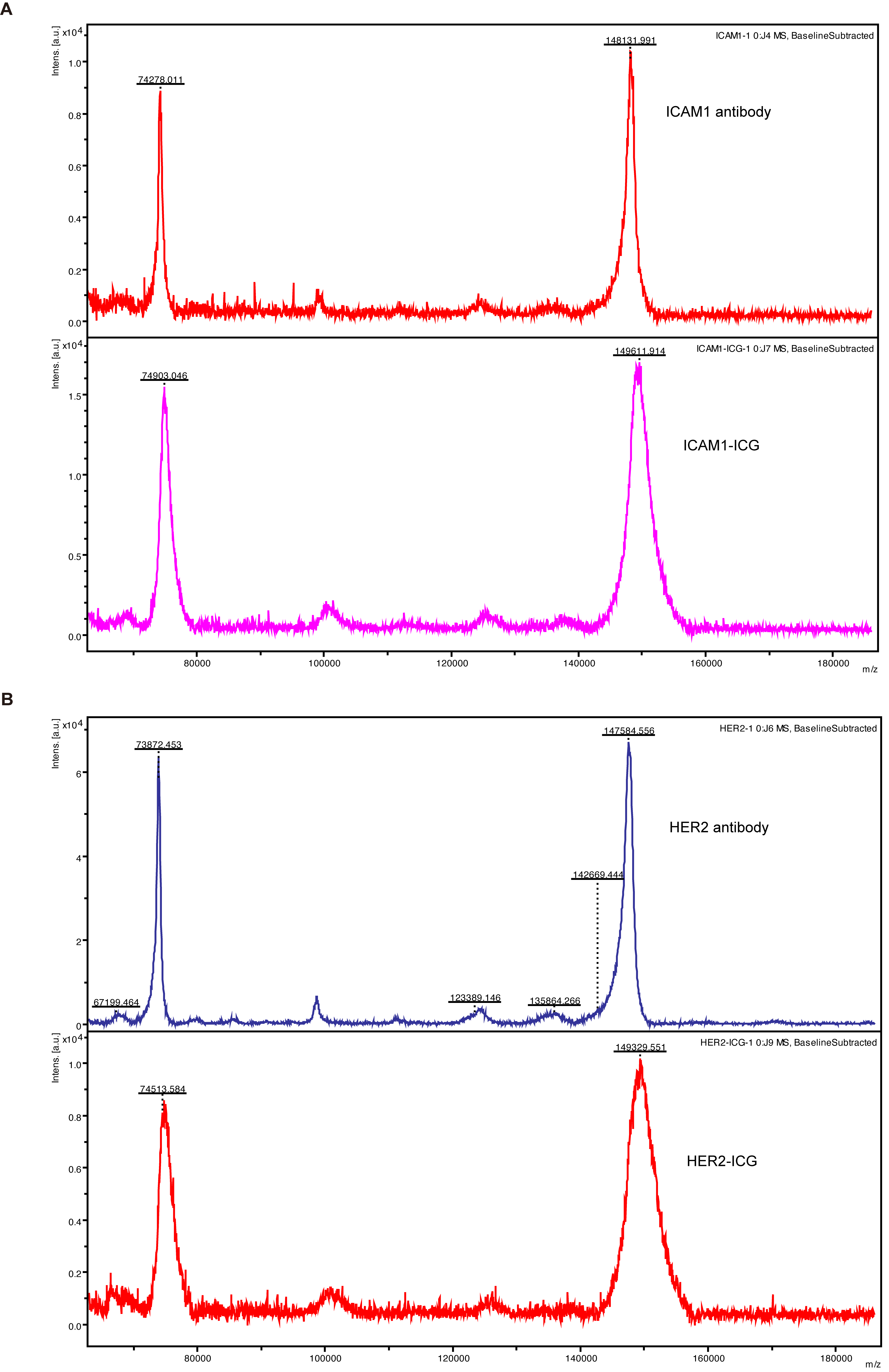
**

**Figure S1.** (A) Mass spectrometry of ICAM1 antibody (top) and ICAM1-ICG (bottom). (B)Mass spectrometry of HER2 antibody (top) and HER2-ICG (bottom).

**
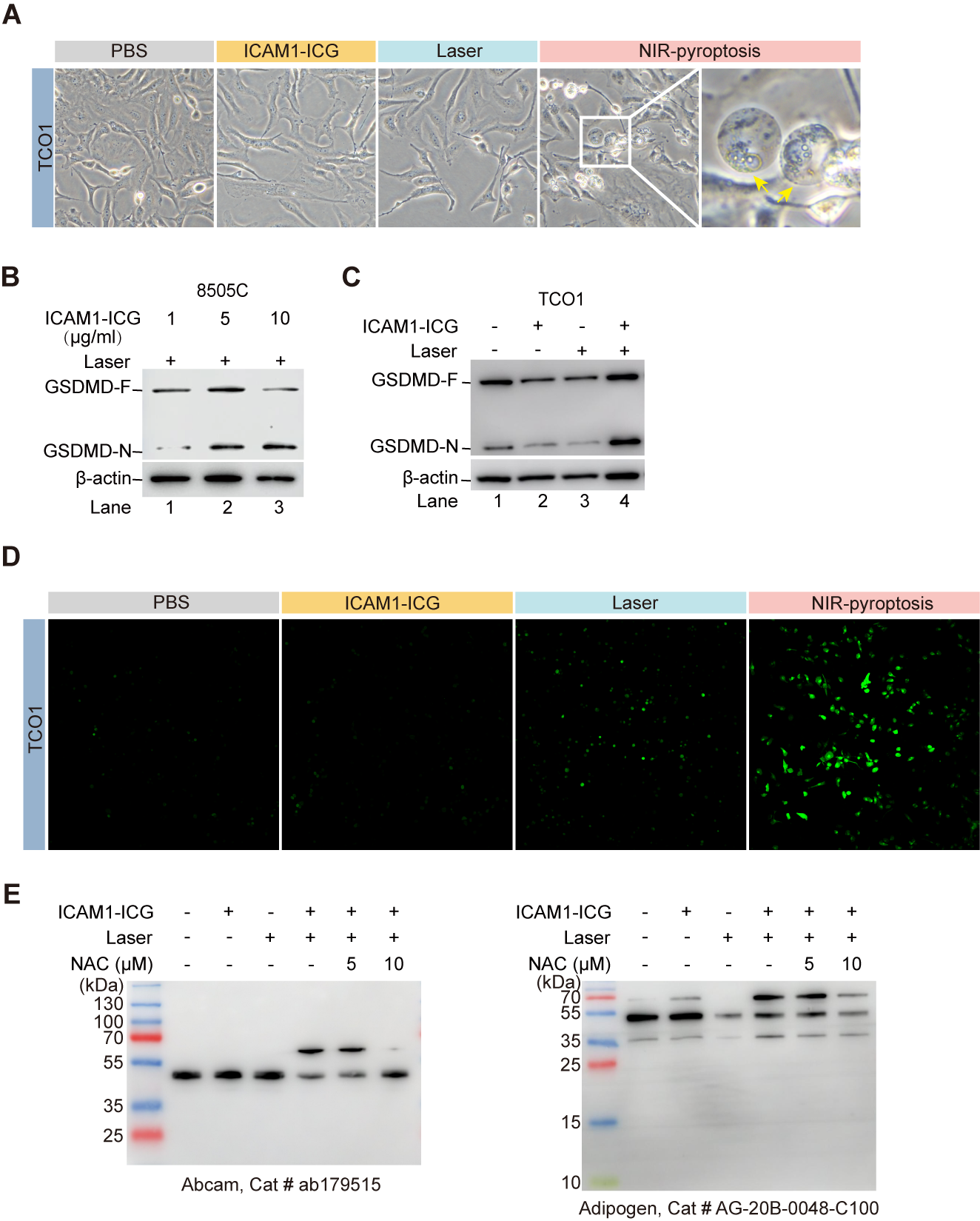
**

**Figure S2.** (A) Representative phase-contrast cell images of ATC cells treated with NIR-pyroptosis. Arrows mark cells that show pyroptotic morphology. (B-C) Western blot analysis of GSDMD cleavage in ATC cells with different treatments. (D) Representative fluorescence images of intracellular ROS detection using DCFH-DA staining. (E) Western blot analysis of caspase-1 cleavage in ATC cells with different treatments.
